# Mechanical Activation of Piezo1 Drives Osteoarthritis Through Kdm5c‐Mediated Epigenetic Silencing

**DOI:** 10.1002/advs.76089

**Published:** 2026-06-12

**Authors:** Tianyou Kan, Xuran Li, Han Wang, Lin Sun, Yao Wang, Jiangdong Wu, Junqi Cui, Yiqi Yang, Kai Yuan, Linyang Chu, Liao Wang, Hanjun Li, Mengning Yan, Zhifeng Yu

**Affiliations:** ^1^ Shanghai Key Laboratory of Orthopedic Implants, Department of Orthopedic Surgery, Shanghai Ninth People's Hospital Shanghai Jiao Tong University School of Medicine Shanghai China; ^2^ Department of Bone and Joint Surgery, Department of Orthopedics Renji Hospital, School of Medicine, Shanghai Jiao Tong University Shanghai China; ^3^ Department of Pathology Shanghai Ninth People's Hospital, Shanghai Jiao Tong University School of Medicine Shanghai China; ^4^ Department of Orthopedics The First Affiliated Hospital, Zhejiang University School of Medicine Hangzhou China; ^5^ Department of Sports Medicine, National Center For Orthopaedics Shanghai Sixth People's Hospital, Shanghai Jiao Tong University School of Medicine Shanghai China; ^6^ Renji‐Med X Clinical Stem Cell Research Center Renji Hospital, Shanghai Jiao Tong University School of Medicine Shanghai China

## Abstract

Mechanical stress is the most important factor affecting the progression of osteoarthritis (OA), but the mechanism linking mechanical stress to transcriptional repression remains elusive. Here, the study finds that mechanical stress induced epigenetic changes that can serve as therapeutic targets for osteoarthritis. By using Piezo1 conditional knockout (*Col2a1^CreERT^; Piezo1^flox/flox^*) mice, it was found that Piezo1 activation by excessive mechanical stress can trigger chromatin remodeling via cytoskeletal force transmission, promoting the histone demethylase Kdm5c‐mediated epigenetic silencing. Kdm5c in turn erases H3K4me3 marks from promoters of cartilage‐anabolic genes *Col2a1* and *Runx3*, silencing their expression. Genetic ablation of Kdm5c rescues mechanical stress‐induced cartilage degradation. Through drug repurposing, the study identifies telmisartan as a direct Kdm5c inhibitor that blocks this pathway and demonstrates disease‐modifying efficacy in mouse OA models and human cartilage explants. These results establish the Piezo1–Kdm5c axis as a fundamental driver of OA and position telmisartan as a mechano‐epigenetic therapy with immediate translational potential.

## Graphical abstract

Excessive mechanical stress activates Piezo1, triggering Ca^2^^+^‐dependent cytoskeletal forces that deform the nucleus and reduce H3K4me3. Kdm5c demethylates H3K4me3 at *Col2a1* and *Runx3* promoters. Kdm5c knockout rescues degradation. Repurposed telmisartan directly inhibits Kdm5c, blocking this axis and showing disease‐modifying efficacy in mouse OA models and human explants, with immediate translational potential.

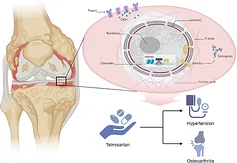

## 1 Introduction

Osteoarthritis (OA) is a highly prevalent and debilitating joint disorder, pathologically characterized by the progressive destruction of articular cartilage, subchondral bone sclerosis, and synovitis [1]. Among the multifactorial drivers of OA, mechanical stress is the most important factor affecting the progression of osteoarthritis [2]. However, a central and unresolved paradox remains: how is mechanical stress converted into stable, long‐term alterations in gene expression that perpetuate cartilage degradation? This critical knowledge gap severely impedes the development of targeted, mechanism‐based therapeutics [2, 3].

As the sole cellular residents of articular cartilage, chondrocytes are entrusted with maintaining extracellular matrix (ECM) homeostasis [4]. They are embedded within a complex ECM and persistently perceive mechanical signals transmitted through this matrix through ion channels including Piezo1 [5, 6]. Substantial evidence confirms that Piezo1 is activated and upregulated in OA cartilage, and its inhibition can attenuate disease progression [7, 8]. However, Piezo1 also plays indispensable roles in physiological cartilage development and homeostasis [5, 9]. This functional duality presents a significant therapeutic challenge that global suppression of Piezo1 may disrupt vital physiological processes, which underscoring the compelling need to move “downstream” and identify the specific pathological effectors that transduce aberrant Piezo1 signaling into cartilage destruction [9, 10].

Mechanical signals can transmit into the nucleus, which is now recognized as a central mechanosensory organelle [11, 12]. Mechanical stress can induce nuclear deformation [13, 14], alter the mechanical properties of the nuclear lamina [15], and ultimately lead to chromatin reorganization and changes in gene expression [11, 16]. This raises a compelling hypothesis: pathological mechanotransduction might converge on the nucleus to instigate a maladaptive epigenetic reprogramming of the chondrocyte during OA progression. Epigenetic alterations are increasingly implicated in OA pathogenesis [17]. Dysregulated histone methylation represents a major regulatory mechanism, with changes in H3K9me3, H3K27me3, H3K36me3 and H3K79me3 all being linked to disrupted chondrocyte function [18, 19, 20, 21]. While these modifications are known to respond to biochemical stimuli, but emerging evidence indicates they can also be directly regulated by mechanical stress [22, 23, 24]. Nevertheless, the precise mechanism by which mechanical stress dictates the histone methylation landscape in OA chondrocytes remains largely undefined. Notably, the potential erosion of H3K4me3 by mechanical stress has been largely overlooked in OA epigenetics. As a quintessential chromatin mark for active transcription and a putative regulator of cellular “youthfulness” [19], its absence from the research landscape represents a critical knowledge gap.

Here, we hypothesize that Piezo1 activation drives a downstream cascade that compromises nuclear envelope integrity, thereby facilitating the epigenetic silencing of key cartilage‐anabolic genes. We aimed to elucidate whether this pathway involves mechanical stress induced nuclear pore dilation and the subsequent nuclear import of the H3K4me3‐erasing enzyme Kdm5c, and leverage this mechanistic insight for therapy by repurposing an existing clinical drug to block Piezo1‐Kdm5c mechano‐epigenetic axis, thereby offering a precise and translatable strategy for modifying OA progression.

## 2 Results

### 2.1 Mechanical Stress Induces Piezo1 Upregulation and Nuclear Deformation in Osteoarthritis

**FIGURE 1:**
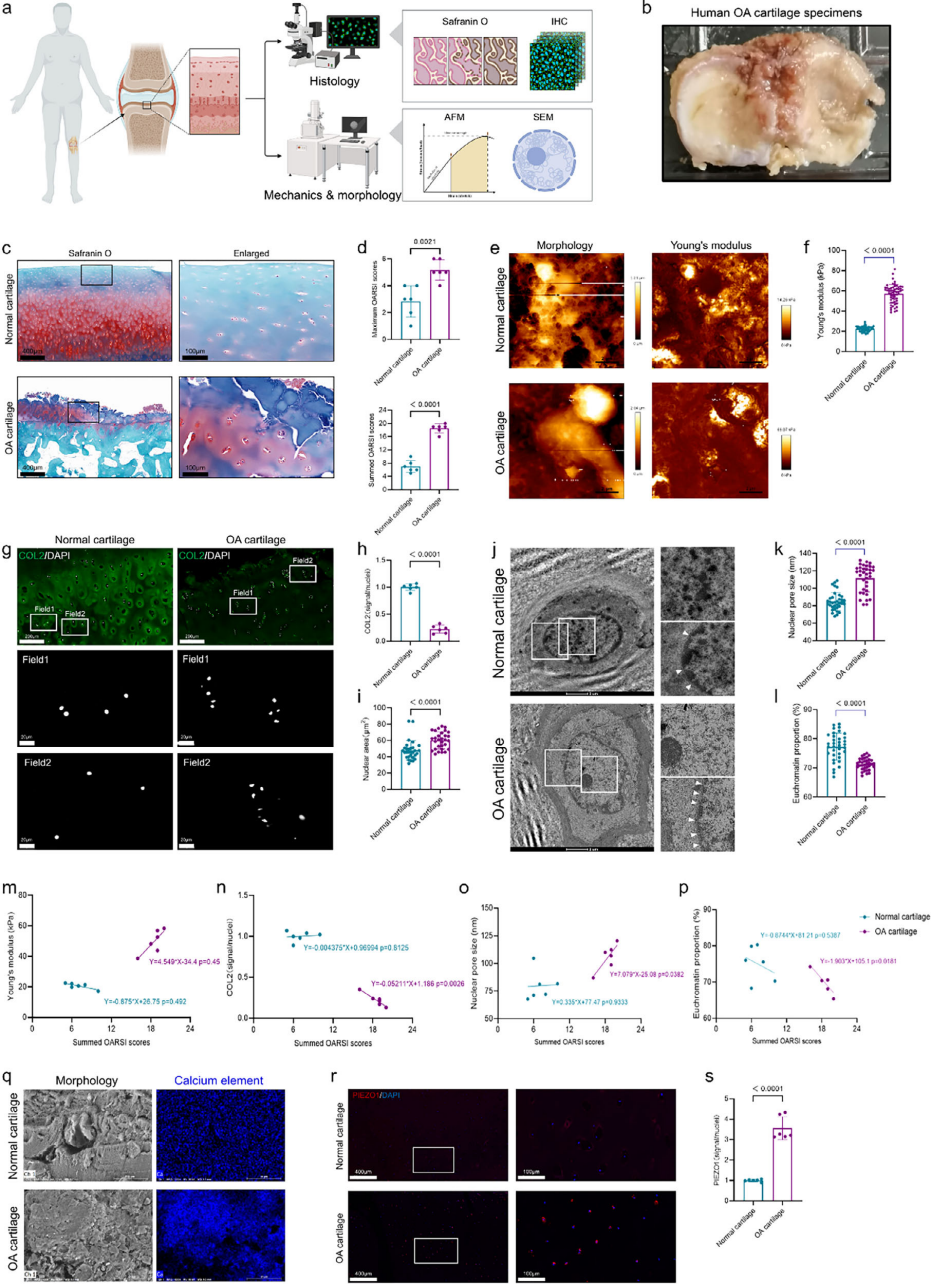
Mechanical stress induces Piezo1 upregulation and nuclear deformation in osteoarthritis. (a) Schematic diagram of OA clinical specimen analysis process. (b) General photos of OA clinical specimens. (c) Representative Safranin O staining of human OA cartilage samples from the unstressed side and the stressed side. Scale bars: 400 and 100 µm. (d) Maximum and summed OARSI scores of OA cartilage sample (*n* = 6). (e) AFM morphology and mechanical scanning heatmaps of the surface from OA cartilage samples from the unstressed side and the stressed side. Scale bars: 2 µm. (f) Young's modulus of OA cartilage quantified by AFM from the unstressed side and the stressed side (*n* = 60 samples from six human OA samples). (g) Immunofluorescence of COL2 and DAPI of OA cartilage samples from the unstressed side and the stressed side. Scale bars: 200 and 20 µm. (h) Quantification of COL2 fluorescence intensity of OA cartilage samples from the unstressed side and the stressed side (*n* = 6). (i) Chondrocyte nuclear area of OA cartilage samples from the unstressed side and the stressed side (*n* = 30 samples from six human OA samples). (j) TEM images of OA cartilage samples. (k) The nuclear pore size of OA cartilage samples (from 35 cells of each group). (l) The proportion of euchromatin of OA cartilage samples (from 35 cells of each group). (m) Correlation analysis of OARSI score and Young's modulus of OA cartilage samples (*n* = 6). (n) Correlation analysis of OARSI score and COL2 expression level of OA cartilage samples (*n* = 6). (o) Correlation analysis of OARSI score and the nuclear pore size of OA cartilage samples (*n* = 6). (p) Correlation analysis of OARSI score and the proportion of euchromatin of OA cartilage samples (*n* = 6). (q) SEM morphology and elemental mapping of Ca in the unstressed side and the stressed side. (r) Immunofluorescence of PIEZO1 and DAPI in human OA cartilage of the unstressed side and the stressed side. Scale bars: 400 and 100 µm. (s) The positive proportions of PIEZO1 positive cells of OA cartilage samples from the unstressed side and the stressed side (*n* = 6). The p value is indicated on the statistical graph. Values are means ± SDs. Comparison between two groups was performed by a two‐tailed Student's *t*‐test.

To investigate the detrimental effects of mechanical stress on cartilage degeneration, we first collected cartilage specimens from patients with OA and varus deformity (Figure 1a,b). Safranin O‐fast green staining revealed more severe wear, thinning, and surface fissures in cartilage from stress‐concentrated side compared to the contralateral side (Figure 1c,d). Atomic force microscopy further demonstrated that aberrant mechanical stress resulted in an uneven cartilage surface with heterogeneous modulus distribution, indicating that chondrocytes reside in a mechanically heterogeneous microenvironment (Figure 1e,f). Immunofluorescence staining COL2 and nuclei showed altered nuclear morphology in OA chondrocytes (Figure 1g,h), characterized by nuclear enlargement (Figure 1i). TEM revealed microstructural changes in nuclei of chondrocytes from articular cartilage on the stress‐concentrated side, including altered chromatin organization and nuclear pore architecture (Figure 1j–l). To determine the association between cartilage injury and mechanical and nuclear morphology, we found that the severity of cartilage damage positively correlated with increased matrix stiffness (Figure 1m) and reduction of COL2 (Figure 1n). Chondrocytes from more severely damaged regions exhibited increased nuclear pore opening and reduced euchromatin density (Figure 1o,p).

After establishing the correlation between OA, mechanics, and nuclear morphology, we hypothesized that these changes were driven by mechanical stress. Scanning electron microscopy with energy‐dispersive X‐ray spectroscopy confirmed that adverse mechanical stress caused significant calcium deposition on the articular cartilage surface of the stress‐concentrated side, concomitant with decreased organic elements (Figure 1q). Furthermore, Piezo1 expression was significantly upregulated in OA chondrocytes (Figure 1r,s). To validate this finding, we established an OA mouse model via DMM surgery to simulate medial tibiofemoral joint cartilage injury (Figure S1a,b). Eight weeks post‐surgery, the articular cartilage of DMM mice underwent surface hardening (Figure S1c,d) and chondrocyte nuclear morphological changes (Figure S1e–g), like human OA cartilage. Piezo1 expression was elevated in mouse OA cartilage (Figure S1h,i) and correlated positively with the degree of nuclear deformation, suggesting an association between mechanical stress, Piezo1 upregulation, and nuclear deformation during OA progression.

### 2.2 Piezo1 Transmits Mechanical Stress to the Nucleus Through Cytoskeletal Rearrangement in Chondrocytes

**FIGURE 2:**
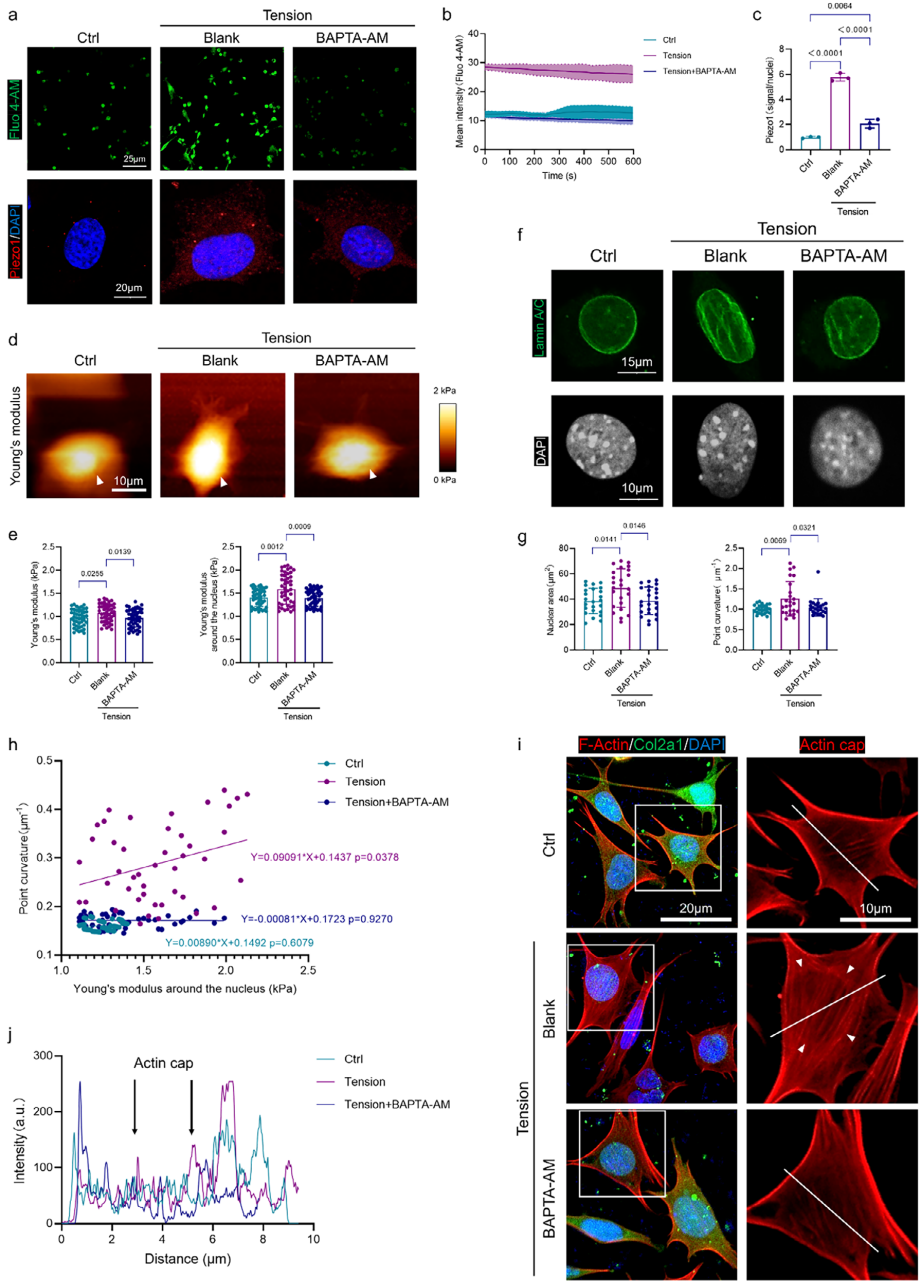
Tensile strain activates mechanotransduction signaling in ATDC5 chondrocytes through Piezo1‐Ca^2+^. (a) Representative Fluo4‐AM fluorescence images and immunofluorescence of Piezo1 and DAPI in Ctrl, Tension and Tension + BAPTA‐AM groups. Scale bars: 25 and 20 µm. (b) Quantification of calcium influx intensity in Ctrl, Tension and Tension + BAPTA‐AM groups within 600 s. (c) Quantification of Piezo1 fluorescence intensity of Ctrl, Tension and Tension + BAPTA‐AM groups (*n* = 3). (d) Atomic force microscopy young's modulus maps of ATDC5 cells of Ctrl, Tension and Tension + BAPTA‐AM groups. Scale bars: 10 µm. (e) Young's modulus of whole cells and young's modulus around the nucleus of ATDC5 cells of Ctrl, Tension and Tension + BAPTA‐AM groups. (*n* = 45). (f) Immunofluorescence of LaminA/C and DAPI of Ctrl and Tension groups. Scale bars: 15 and 10 µm. (g) Nuclear cross‐sectional area and point curvature of nucleus in ATDC5 cells of Ctrl, Tension and Tension + BAPTA‐AM groups (*n* = 24). (h) Correlation analysis of young's modulus around the nucleus and point curvature of Ctrl, Tension and Tension + BAPTA‐AM groups (*n* = 45). (i) Immunofluorescence of F‐Actin, Col2a1 and DAPI of Ctrl, Tension and Tension + BAPTA‐AM groups. Scale bars: 20 and 10 µm. (j) Fluorescence co‐localization analysis of F‐Actin of Ctrl, Tension and Tension + BAPTA‐AM groups. The *p* value is indicated on the statistical graph. Values are means ± SDs. Multiple comparison was performed by one‐way ANOVA with Tukey's post‐hoc analysis.

To elucidate the mechanotransduction pathway by which mechanical stress activates Piezo1 and leads to nuclear aberration, we established an in vitro model of mechanically induced OA by applying cyclic tensile strain (0.5 Hz, 15%) to ATDC5 cells for 12 h. To investigate whether the activation of Piezo1 is mediated by Ca^2+^ for the subsequent mechanotransduction, BAPTA‐AM was also used to remove intracellular Ca^2+^. Using Fluo‐4 AM calcium imaging, we observed a significant and sustained increase in intracellular Ca^2+^ levels following mechanical stimulation. The use of BAPTA‐AM significantly inhibited the signal of Fluo‐4 AM, which demonstrated that it could remove Ca^2+^ from the cells under mechanical loading (Figure 2a,b). Piezo1 expression was upregulated under tensile strain, while BAPTA‐AM simultaneously inhibited the Piezo1 activation induced by mechanical loading (Figure 2a,c). To evaluate nuclear mechanical responses, ATDC5 cells were analyzed via atomic force microscopy before and after mechanical stress. After mechanical loading, overall ATDC5 cells stiffness increased, with a particularly pronounced stiffening effect in the perinuclear region. Similarly, the use of BAPTA‐AM prevented the hardening of ATDC5 cells after mechanical loading (Figure 2d,e). Immunofluorescence staining of Lamin A/C showed that tensile strain not only increased Lamin A/C expression but also induced nuclear shrinkage, followed by an increase in nuclear spread area and curvature (Figure 2f). In summary, mechanical stress caused nuclei to become enlarged and deformed. The application of BAPTA‐AM further prevented the occurrence of nuclear deformation. (Figure 2g). We further assessed correlations between cellular biomechanical properties and nuclear morphology. The perinuclear stiffness showed a strong positive correlation with nuclear curvature in the mechanically stimulated group (Figure 2h). Concomitantly, mechanical stress significantly suppressed Col2a1 expression and induced extensive cytoskeleton rearrangement. Specifically, after the ATDC5 cells were subjected to mechanical stress, the actin filaments arranged along the tensile stress direction on one hand, and gathered around the cell nucleus on the other hand. However, the changes in the actin filaments after the force application disappeared after the use of BAPTA‐AM (Figure 2i,j). In conclusion, these results indicate that tension strain activates Piezo1‐ Ca^2+^, which then trigger mechanotransduction mediated by the cytoskeleton to be transmitted to the nucleus, thereby causing abnormal mechanical responses and structural changes in the nucleus.

### 2.3 Piezo1 Activation Drives Nuclear Pore Opening and Chromatin Remodeling in Mechanically Loaded Chondrocytes

**FIGURE 3:**
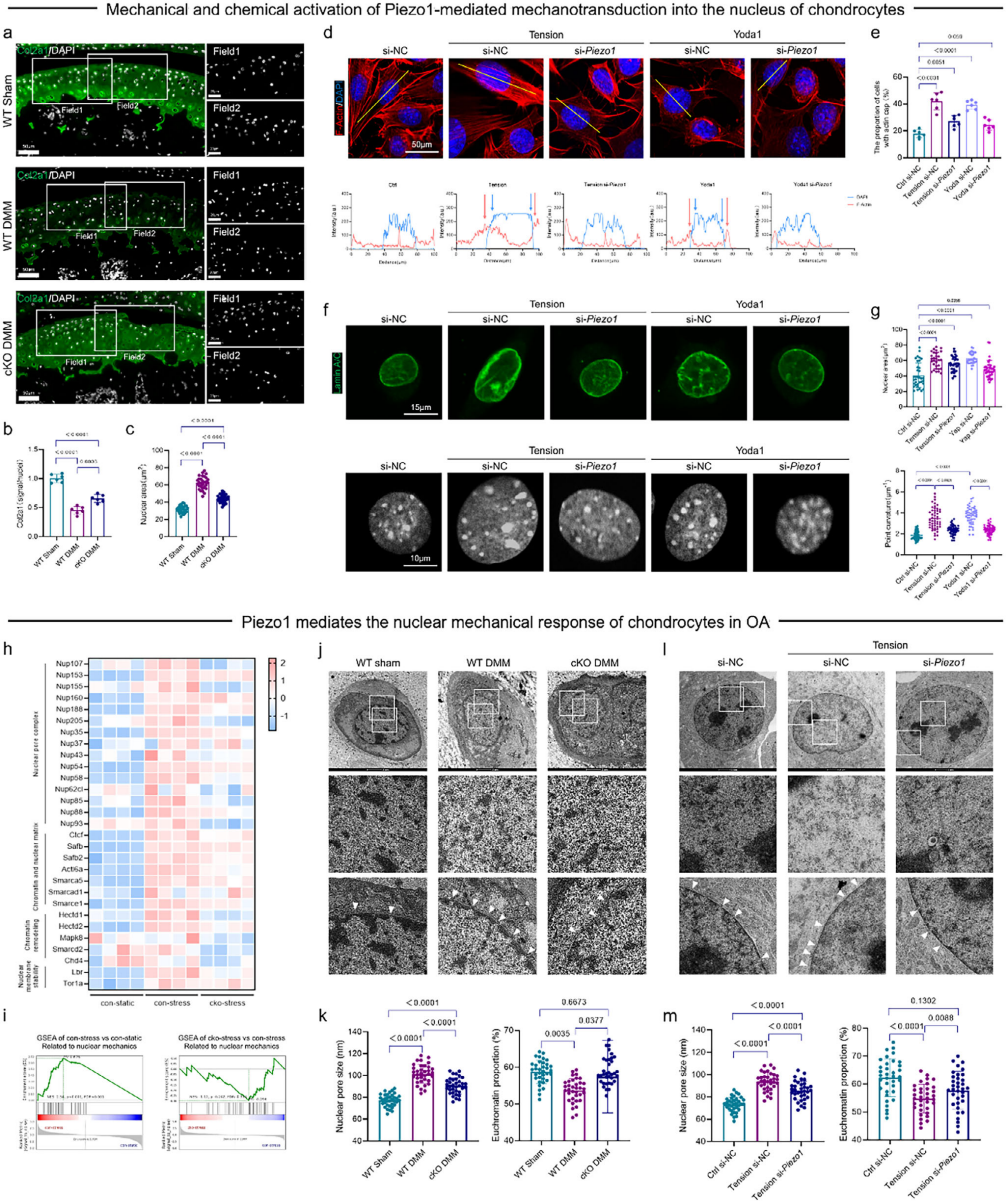
Piezo1 mediates nuclear mechanotransduction in osteoarthritis. (a) Immunofluorescence of Col2a1 and DAPI of WT Sham, WT DMM and cKO DMM groups. Scale bars: 50 and 20 µm. (b) Quantification of COL2 fluorescence intensity of WT Sham, WT DMM and cKO DMM groups (*n* = 6). (c) Chondrocyte nuclear cross‐sectional area of OA cartilage samples of COL2 of WT Sham, WT DMM and cKO DMM groups (*n* = 30 samples from six WT Sham, WT DMM or cKO DMM mice). (d) Immunofluorescence and co‐localization analysis of F‐Actin and DAPI of si‐NC Ctrl, si‐NC Tension, si‐*Piezo1* Tension, si‐NC Yoda1 and si‐*Piezo1* Yoda1 groups. Scale bars: 50 µm. (e) Percentage of cells with perinuclear actin cap of si‐NC Ctrl, si‐NC Tension, si‐*Piezo1* Tension, si‐NC Yoda1 and si‐*Piezo1* Yoda1 groups (*n* = 6). (f) Immunofluorescence of LaminA/C and DAPI of si‐NC Ctrl, si‐NC Tension, si‐*Piezo1* Tension, si‐NC Yoda1 and si‐*Piezo1* Yoda1 groups. Scale bars: 15 and 10 µm. (g) Nuclear area of ATDC5 cells of si‐NC Ctrl, si‐NC Tension, si‐*Piezo1* Tension, si‐NC Yoda1 and si‐*Piezo1* Yoda1 groups (*n* = 35 in si‐NC Ctrl group, *n* = 30 in si‐NC Tension group, *n* = 36 in si‐*Piezo1* Tension group, *n* = 29 in si‐NC Yoda1 group and *n* = 35 in si‐*Piezo1* Yoda1 group). And point curvature of si‐NC Ctrl, si‐NC Tension, si‐*Piezo1* Tension, si‐NC Yoda1 and si‐*Piezo1* Yoda1 groups (from 24 cells per group). (h) Heatmaps of nuclear mechanics related genes expression in con‐static, con‐stress and cko‐stress groups (*n* = 4). (i) GSEA analysis of nuclear mechanics related pathways in con‐static, con‐stress and cko‐stress groups. (j) TEM images of chondrocytes nuclei in WT sham, WT DMM and cKO DMM groups. (k) Quantification of nuclear pore size and the proportion of euchromatin of chondrocytes in WT sham, WT DMM and cKO DMM groups (from 35 cells per group). (l) TEM images of chondrocytes nuclei in si‐NC Ctrl, si‐NC Tension and si‐*Piezo1* Tension groups. (m) Quantification of nuclear pore size and euchromatin ratio of chondrocytes in si‐NC Ctrl, si‐NC Tension and si‐*Piezo1* Tension groups (from 35 cells per group). The *p* value is indicated on the statistical graph. Values are means ± SDs. Multiple comparison was performed by one‐way ANOVA with Tukey's post‐hoc analysis.

To investigate the role of Piezo1 in mechanical load‐induced chondrocyte nuclear aberration, we knocked down Piezo1 in chondrocytes using siRNA (Figure S2a,b) and generated chondrocyte‐specific Piezo1 knockout (*Col2a1^CreERT^; Piezo1^flox/flox^*, cKO) mice. OA was induced by DMM surgery (Figure S2c). Histological and immunofluorescence analyses performed 8 weeks post‐surgery showed that Piezo1 deletion in chondrocytes alleviated articular cartilage damage and nuclear deformation (Figure 3a–c; Figure S2d,e). Importantly, Piezo1 deficiency ameliorated nuclear aberrations. Mechanical stimulation (tensile strain) or chemical activation of Piezo1 with Yoda1 induced cytoskeletal rearrangement and promoted the formation of an actin ring. This effect was abolished upon Piezo1 knockdown (Figure 3d,e; Figure S2f,g). Both mechanical and chemical activation of Piezo1 caused force transmission to the nucleus, leading to enrichment and shrinkage of nuclear membrane protein Lamin A/C, indicative of intense nuclear mechanical stimulation. Piezo1 loss prevented these changes (Figure 3f). Consistent with our in vitro findings, Piezo1 activation increased chondrocyte nuclear size and curvature, confirming its essential role in loading‐induced nuclear deformation (Figure 3f,g). In conclusion, Piezo1 is a key mechanotransduction factor causing mechanical load‐induced nuclear aberration in chondrocytes. Interfering with its expression reverses nuclear aberration and improves cartilage homeostasis.

RNA sequencing revealed that mechanical stress upregulated genes related to nuclear pore complexes, chromatin remodeling, and nuclear membrane stability. Most of these changes were attenuated in Piezo1 knockout chondrocytes (Figure 3h,i), suggesting Piezo1 mediates nuclear structural alterations that may facilitate chromatin remodeling and nuclear pore opening. Histological and ultrastructural analysis of mouse articular cartilage and in vitro cells confirmed that DMM surgery reduced chromatin density and promoted nuclear pores opening. These effects were partially reversed by Piezo1 deletion (Figure 3j,k). Similarly, tensile strain reduced euchromatin content and increased nuclear pore diameter, both of which were rescued by Piezo1 knockdown (Figure 3l,m). Together, these results demonstrate that mechanical stress activates Piezo1 to induce cytoskeleton‐driven nuclear aberration, nuclear pore opening, and chromatin remodeling, thereby contributing to OA progression.

### 2.4 Mechanical Activation of Piezo1 Upregulates Kdm5c Expression and Reduces H3K4me3 Levels

**FIGURE 4:**
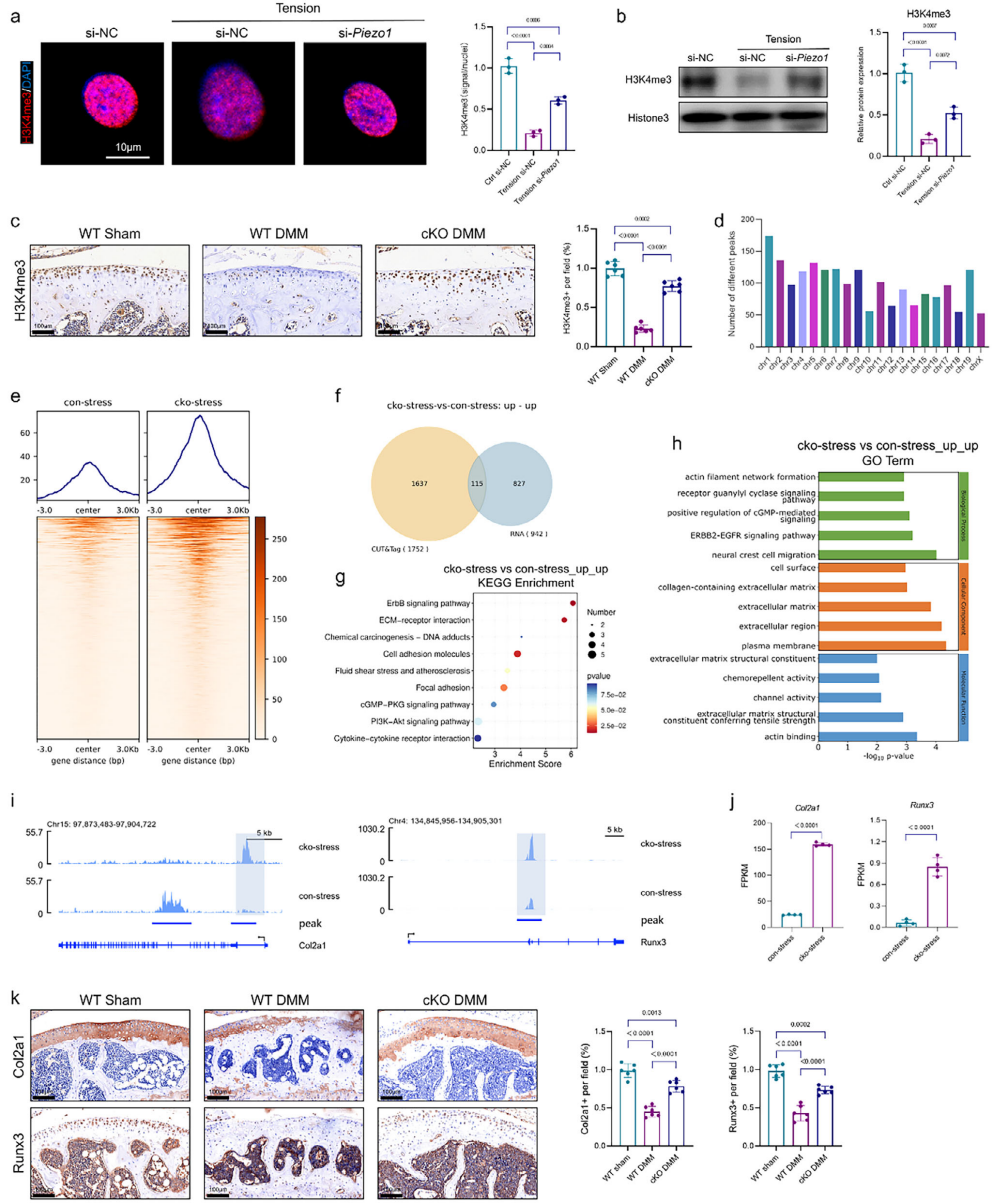
Piezo1 mediates mechanical regulation of H3K4me3 to activate *Col2a1* and *Runx3* transcription in osteoarthritis. (a) Immunofluorescence and relative fluorescence intensity of H3K4me3 and DAPI of si‐NC Ctrl, si‐NC Tension and si‐*Piezo1* Tension groups (*n* = 3). Scale bars: 10 µm. (b) The protein expression levels of H3K4me3 of si‐NC Ctrl, si‐NC Tension and si‐*Piezo1* Tension groups (*n* = 3). (c) Immunohistochemistry and quantification of H3K4me3 positive cells in articular cartilage in WT Sham, WT DMM and cKO DMM groups (*n* = 6). Scale bars: 100 µm. (d) The number of peaks defined in each chromosome of chondrocytes after Piezo1 knockout. (e) Profile of H3K4me3 peak density relative to transcription start sites in the con‐stress and cko‐stress groups. (f) The differentially expressed genes of CUT&Tag and RNA‐seq were simultaneously upregulated in con‐stress and cko‐stress groups. (g) KEGG pathway enrichment analysis of the differentially expressed genes with simultaneous upregulation of CUT&Tag and RNA‐seq in con‐stress and cko‐stress groups. (h) GO term enrichment analysis of the differentially expressed genes with simultaneous upregulation of CUT&Tag and RNA‐seq in con‐stress and cko‐stress groups. (i) Integrative Genomics Viewer tracks showing H3K4me3 enrichment in the *Col2a1* and *Runx3* loci under control and mechanical stress conditions with or without Piezo1 knockout. Representative RefSeq transcripts (*Col2a1*: NM_001113515.2 and *Runx3*: NM_001369050.1) are shown below the signal tracks, with arrows indicating transcription start sites (TSS). The H3K4me3‐enriched regions (marked by blue square frames) are located within the promoter regions (from −2 kb to +0.5 kb relative to the TSS) of these transcripts. Regions of chromatin with altered status are marked with blue square frames. (j) Relative expression levels of *Col2a1* and *Runx3* genes (*n* = 4). (k) Immunohistochemistry and the positive proportions of Col2a1 and Runx3 in WT Sham, WT DMM and cKO DMM groups (*n* = 6). Scale bars: 100 µm. The p value is indicated on the statistical graph. Values are means ± SDs. Multiple comparison was performed by one‐way ANOVA with Tukey's post‐hoc analysis. And comparison between two groups was performed by a two‐tailed Student's *t*‐test.

Chromatin remodeling is often linked to alterations in histone modification. To further explore Piezo1‐mediated epigenetic changes, we investigated methylation modifications at multiple histone H3 sites (H3K4, H3K9, H3K27, H3K36, and H3K79) in chondrocytes under mechanical load (Figure S3a–d). Among these, only H3K4 methylation was significantly altered following mechanical stimulation; however, Piezo1 knockdown did not significantly affect their methylation status. Mechanical stress decreased H3K4me3 levels in chondrocytes, which were rescued by Piezo1 knockdown (Figure 4a,b) and in chondrocyte‐specific Piezo1 cKO mice (Figure 4c). CUT&Tag analysis revealed H3K4me3 was predominantly enriched at promoter regions of chondrocyte genes (Figure S4a–e). Piezo1 knockout under mechanical stress significantly increased the number of H3K4me3‐bound peaks (Figure 4d,e). Integrated transcriptomic and epigenomic analysis revealed that differentially expressed genes were mainly associated with extracellular matrix organization, mechanotransduction, and related signaling pathways (Figure S4f,g). As H3K4me3 is associated with transcriptional activation, we analyzed genes that were both upregulated and epigenetically enriched in H3K4me3 following Piezo1 knockout. These genes were primarily related to cellular mechanical response and nucleocytoplasmic transport functions (Figure 4f–h). Specifically, we observed increased H3K4me3 binding at the promoters of *Runx3* and *Col2a1*, leading to an increase in their transcription (Figure 4i–k). These results demonstrate that mechanical stress activates Piezo1 to upregulate Kdm5c expression at both mRNA and protein levels, which in turn reduces H3K4me3 levels, thereby suppressing the expression of key chondrogenic genes, such as *Runx3* and *Col2a1*, and disrupting cartilage homeostasis.

### 2.5 Kdm5c Serves as Mechanosensitive Epigenetic Checkpoint in Osteoarthritis Pathogenesis

**FIGURE 5:**
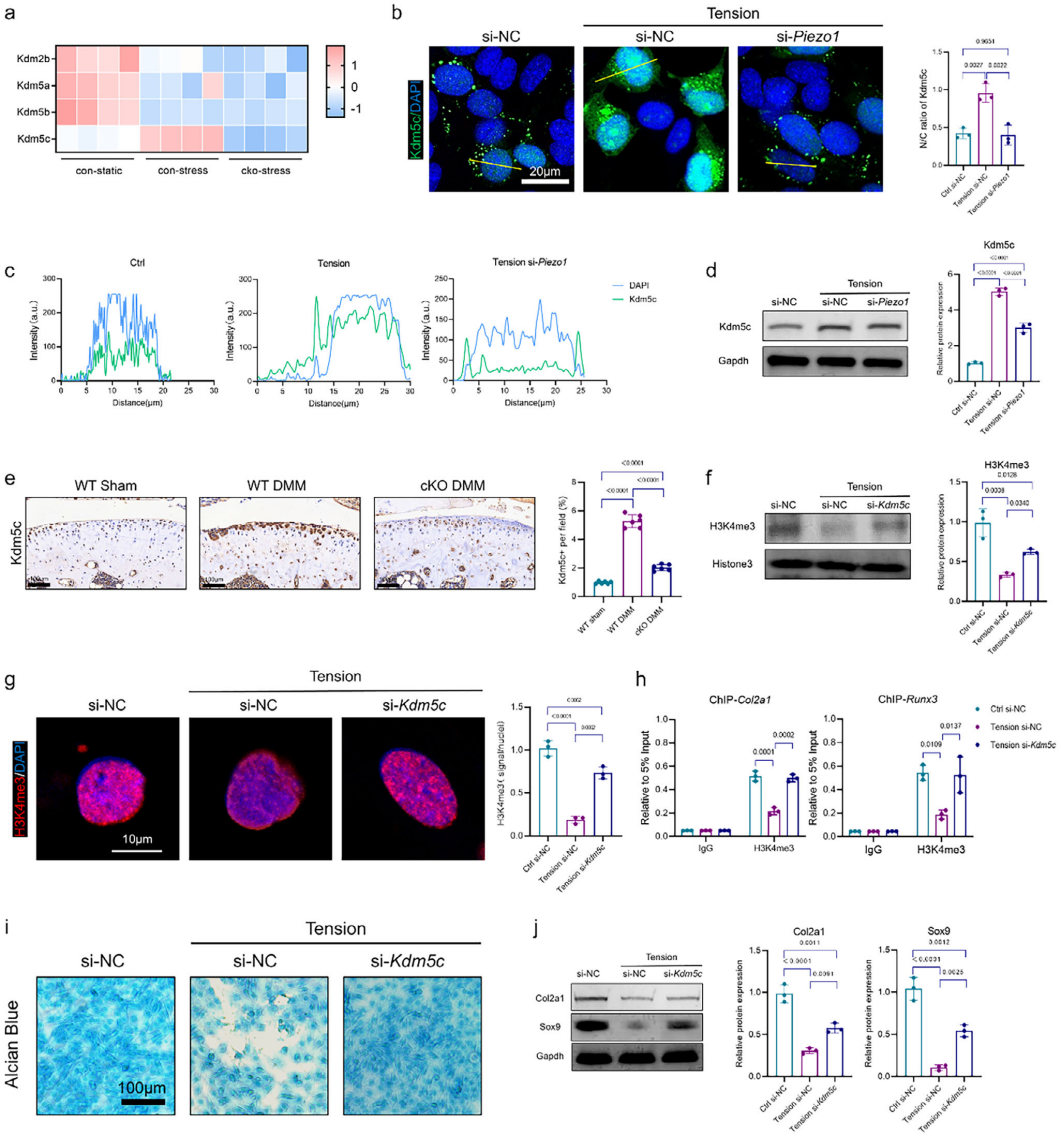
Piezo1‐mediated mechanotransduction promotes Kdm5c activity and suppresses H3K4me3. (a) Heatmap of methylase and demethylase gene expression in con‐static, con‐stress and cko‐stress groups (*n* = 4). (b) Immunofluorescence and relative fluorescence intensity of Kdm5c and DAPI of si‐NC Ctrl, si‐NC Tension and si‐*Piezo1* Tension groups. Scale bars: 20 µm (*n* = 3). (c) Fluorescence co‐localization analysis of Kdm5c and DAPI of si‐NC Ctrl, si‐NC Tension and si‐*Piezo1* Tension groups. (d) The protein expression levels of Kdm5c of si‐NC Ctrl, si‐NC Tension and si‐*Piezo1* Tension groups (*n* = 3). (e) Immunohistochemistry and quantification of Kdm5c positive cells in cartilage from WT Sham, WT DMM and cKO DMM groups (*n* = 6). Scale bars: 100 µm. (f) The protein expression levels of H3K4me3 of si‐NC Ctrl, si‐NC Tension and si‐*Kdm5c* Tension groups (*n* = 3). (g) Immunofluorescence and relative fluorescence intensity of H3K4me3 and DAPI of si‐NC Ctrl, si‐NC Tension and si‐*Kdm5c* Tension groups (n=3). Scale bars: 10 µm. (h) ChIP‐PCR of H3K4me3 on *Col2a1* and *Runx3* in promoters of si‐NC Ctrl, si‐NC Tension and si‐*Kdm5c* Tension groups (*n* = 3). (i) Alcian blue staining of ATDC5 cells of si‐NC Ctrl, si‐NC Tension and si‐*Kdm5c* Tension groups. Scale bars: 100 µm. (j) The protein expression levels of Col2a1, Sox9, Mmp13, and Gapdh of si‐NC Ctrl, si‐NC Tension and si‐*Kdm5c* Tension groups (*n* = 3). The *p* value is indicated on the statistical graph. Values are means ± SDs. Multiple comparison was performed by one‐way ANOVA with Tukey's post‐hoc analysis.

To identify whether mechanical stress regulates H3K4me3‐modifying enzymes through Piezo1, we analyzed transcriptomic data of genes encoding H3K4me3 demethylases. Kdm5c was the only demethylase upregulated under mechanical stress and downregulated following Piezo1 knockout (Figure 5a). Mechanical stress increased Kdm5c expression and Piezo1 knockdown abolished these effects (Figure 5b–d). Altered Kdm5c expression was also observed in Piezo1 knockout mice (Figure 5e).

The siRNA‐mediated Kdm5c knockdown in chondrocytes directly increased global H3K4me3 levels, consistent with its role as an H3K4me3 demethylase (Figure 5f,g). To directly assess whether Kdm5c mediates H3K4me3 loss at target gene promoters, we performed H3K4me3 ChIP‐PCR. Mechanical stress significantly reduced H3K4me3 enrichment at the *Col2a1* and *Runx3* promoters, and this reduction was abolished by Kdm5c knockdown (Figure 5h). Remarkably, Kdm5c loss attenuated mechanical stress‐induced cartilage matrix degradation, as shown by Alcian blue staining (Figure 5i), and restored the expression of cartilage‐protective genes (Figure 5j). These findings establish Kdm5c as a mechanosensitive epigenetic regulator downstream of Piezo1 and highlight its potential as a therapeutic target for restoring cartilage homeostasis in OA.

### 2.6 Telmisartan Inhibits Kdm5c to Attenuate Mechano‐Epigenetic Signaling in Osteoarthritis

**FIGURE 6:**
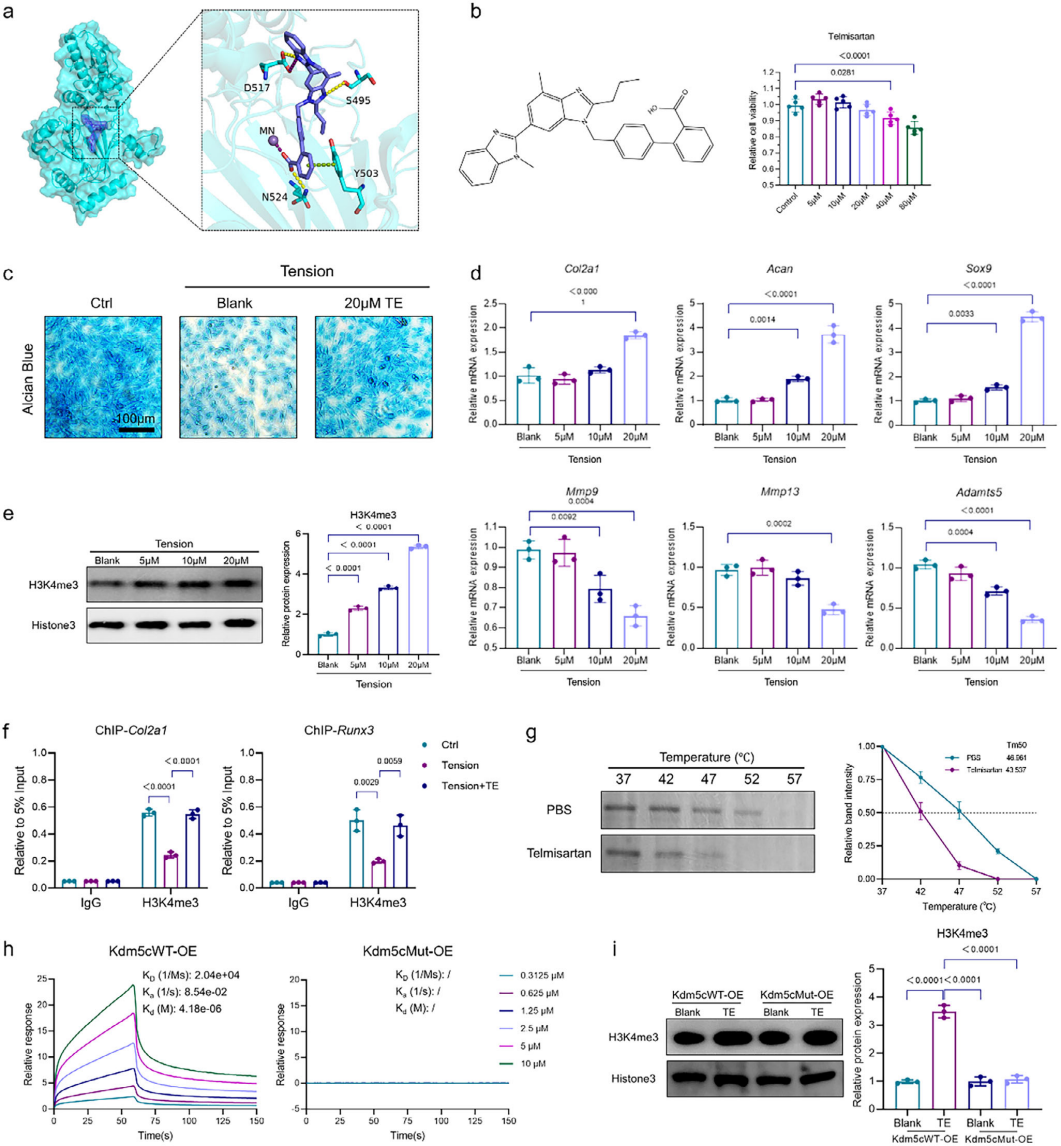
Telmisartan targets Kdm5c to ameliorate mechanical stress induced chondrocyte injury. (a) Schematic diagram of the interaction between telmisartan and the Kdm5c. (b) Chemical structures of the candidate compounds: telmisartan, and Cell viability assay to determine safe concentration ranges of telmisartan (*n* = 3). (c) Alcian blue staining of glycosaminoglycans in mechanically loaded chondrocytes treated with each compound at their maximum safe concentration. (d) Dose‐responsive effect of telmisartan on the mRNA expression levels of *Col2a1*, *Acan*, *Sox9*, *Mmp9*, *Mmp13*, and *Adamts5* in chondrocytes induced by mechanical stress at different concentrations (*n* = 3). (e) The effect of telmisartan on the protein expression levels of H3K4me3 in chondrocytes induced by mechanical stress at different concentrations (*n* = 3). (f) ChIP‐PCR of H3K4me3 on *Col2a1* and *Runx3* in promoters of Ctrl, Tension and Tension + TE groups (*n* = 3). (g) The protein expression levels and the thermal stability curves of Kdm5c in the PBS group and the telmisartan group at different temperatures (*n* =3). (h) SPR verified the binding affinity of telmisartan to Kdm5cWT‐OE and mutant Kdm5cMut‐OE (S495A, D517A, and N524A). (i) The effect of telmisartan on the protein expression levels of H3K4me3 in chondrocytes of Kdm5cWT‐OE and mutant Kdm5cMut‐OE induced by mechanical stress (*n* = 3). The p value is indicated on the statistical graph. Values are means ± SDs. Multiple comparison was performed by one‐way ANOVA with Tukey's post‐hoc analysis.

Having identified chondrocyte Kdm5c as a potential therapeutic target in OA, we performed molecular docking‐based screening of a clinical drug library to identify developable Kdm5c inhibitors (Figure S5a–d). Toxicity assessments revealed that telmisartan exhibited lower cytotoxicity than other top candidate compounds (Figure S6a,b), supporting its selection for further evaluation. Molecular docking confirmed that telmisartan stably binds within the active pocket structure of Kdm5c (Figure 6a). We determined that telmisartan was safe for chondrocytes at concentrations up to 20 µM (Figure 6b).

Within the maximum safe concentration range, telmisartan effectively protected against mechanically induced cartilage damage in vitro in a dose‐dependent manner (Figure 6c,d). Telmisartan increases H3K4me3 levels at different concentrations, consistent with Kdm5c inhibition (Figure 6e). To assess whether telmisartan treatment leads to the deletion of H3K4me3 at the target gene promoter, we conducted a ChIP‐PCR for H3K4me3. Mechanical stress significantly reduced the enrichment level of H3K4me3 at the promoters of *Col2a1* and *Runx3*, and this reduction was eliminated after the addition of telmisartan (Figure 6f). H3K4me3 restoration after telmisartan treatment suggests inhibition of Kdm5c enzymatic activity at these promoters, although whether Kdm5c remains bound or is displaced cannot be distinguished by this assay. Furthermore, telmisartan reduced Kdm5c protein stability under thermal challenge, supporting its role as a functional inhibitor (Figure 6g). To validate the predicted binding sites, we generated a triple‐point mutant of Kdm5c (S495A/D517A/N524A). SPR analysis revealed that while wild‐type Kdm5c bound telmisartan with a K_D_ of 4.18 × 10^−^^6^ M, the mutant Kdm5c showed markedly reduced binding affinity (Figure 6h). Furthermore, we established ATDC5 cell lines stably expressing the Kdm5c mutant. In wild‐type chondrocytes, telmisartan effectively restored H3K4me3 levels under mechanical stress, whereas in mutant Kdm5c‐expressing cells, the protective effect of telmisartan on H3K4me3 was largely abrogated (Figure 6i). Therefore, after the Kdm5c mutation, telmisartan lost its ability to rescue mechanical‐induced cartilage damage (Figure S7a,b). These results confirm that telmisartan directly binds to Kdm5c at the predicted residues to exert its functional inhibition.

### 2.7 Telmisartan Exhibits Disease‐Modifying Effects in Mice and Human Osteoarthritis Models via Epigenetic Mechanism

**FIGURE 7:**
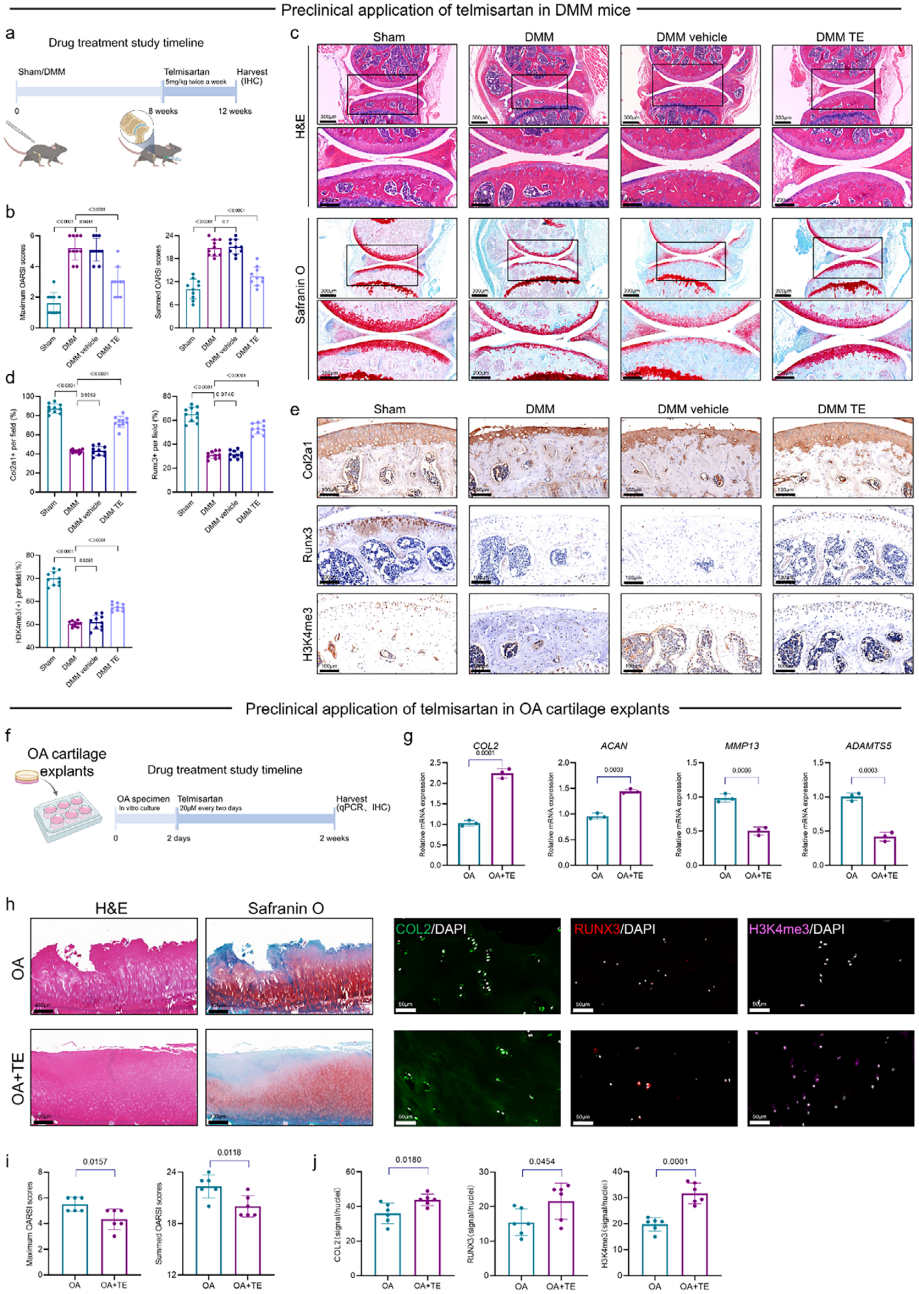
Telmisartan ameliorates osteoarthritis by targeting Kdm5c in mice and human cartilage explants. (a) Workflow of telmisartan treatment in DMM mice. (b) Maximum OARSI scores and summed OARSI scores of Sham, DMM, DMM vehicle and DMM TE groups (*n* = 10). (c) Representative H&E and safranin O staining of Sham, DMM, DMM vehicle and DMM TE groups. Scale bars: 300 and 200 µm. (d) Quantitative immunohistochemistry analysis of Col2a1, Runx3, and H3K4me3 of Sham, DMM, DMM vehicle and DMM TE groups (*n* = 10). (e) Immunohistochemical staining of Col2a1, Runx3 and H3K4me3 of Sham, DMM, DMM vehicle and DMM TE groups. Scale bars: 100 µm. (f) Experimental workflow of telmisartan treatment in human cartilage explants. (g) The mRNA expression levels of *COL2*, *ACAN*, *MMP13*, and *ADAMTS5* in OA and OA+TE human cartilage explants (before and after telmisartan treatment) (*n* = 3). (h) H&E and safranin O staining, and immunofluorescence of COL2, RUNX3, H3K4me3, and DAPI of OA and OA+TE human cartilage explants. Scale bars: 400 and 50 µm. (i) Maximum OARSI scores and summed OARSI scores of OA and OA+TE human cartilage explants. (*n* = 6). (j) Immunofluorescence of COL2, RUNX3, H3K4me3 and DAPI of OA and OA+TE human cartilage explants (*n* = 6). The *p* value is indicated on the statistical graph. Values are means ± SDs. Multiple comparison was performed by one‐way ANOVA with Tukey's post‐hoc analysis. And comparison between two groups was performed by a two‐tailed Student's *t*‐test.

To assess the therapeutic potential of telmisartan in vivo, we utilized the DMM mouse model. Treatment was initiated 8 weeks after surgery; DMM+TE mice received intra‐articular injections of telmisartan (5 mg kg^−1^) and DMM+Vehicle mice received an equivalent volume of DMSO as a control. Knee joints were harvested at 12 weeks post‐DMM for analysis (Figure 7a). Histological evaluation through H&E and safranin O‐fast green staining, along with OARSI scoring, revealed that telmisartan significantly ameliorated cartilage damage and reduced OARSI scores compared to both DMM and vehicle‐treated groups (Figure 7b,c).

Mechanistically, telmisartan inhibited Kdm5c activity, which correlated with elevated expression of *Col2a1* and *Runx3*, and increased H3K4me3 enrichment at their respective gene promoters in the cartilage of DMM+TE mice (Figure 7d,e). To explore the clinical potential of telmisartan, human OA cartilage explants were cultured ex vivo for 2 weeks (Figure 7f). Telmisartan treatment upregulated expression of matrix synthesis genes (*COL2*, *ACAN*) and downregulated matrix degradation genes (*MMP13*, *ADAMTS5*), indicating a restored anabolic‐catabolic balance (Figure 7g). Furthermore, telmisartan‐treated explants exhibited improved surface integrity and quantitative analysis of human OA cartilage explants confirmed that telmisartan treatment significantly reduced OARSI scores and increased COL2, RUNX3, and H3K4me3 protein levels (Figure 7h–j). These results confirm the disease‐modifying efficacy of telmisartan across species through targeting the mechano‐epigenetic pathway, supporting its potential for clinical application in OA treatment.

## 3 Discussion

**FIGURE 8:**
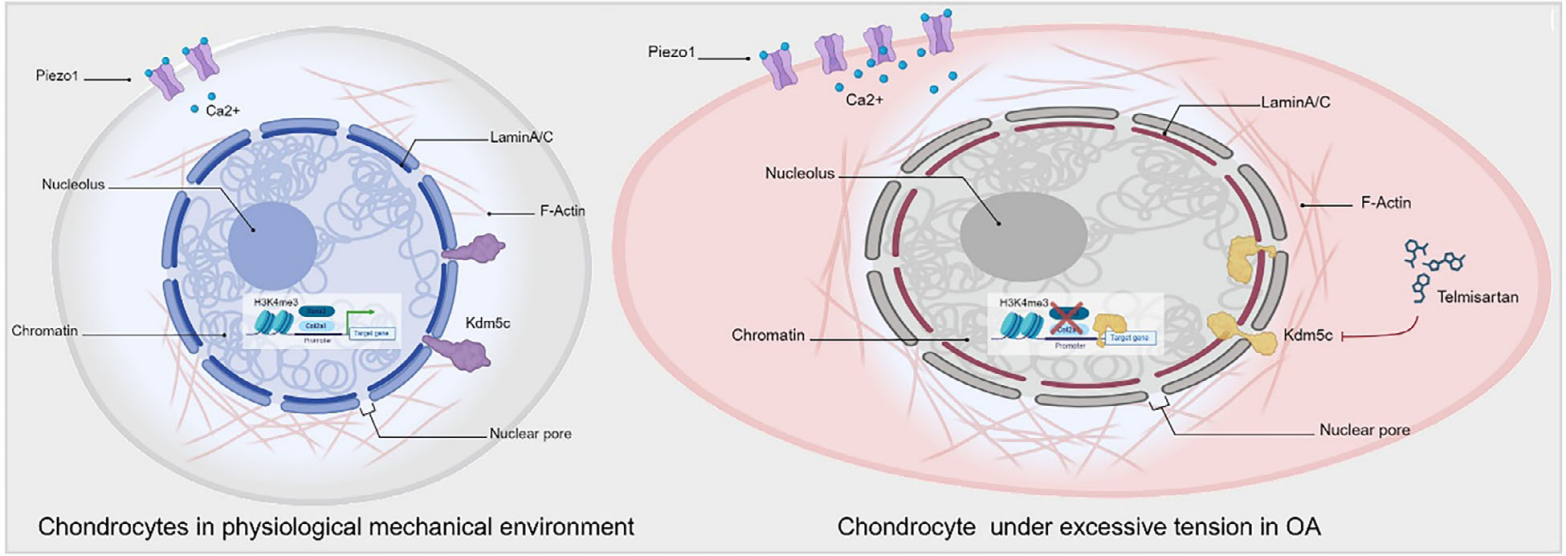
Mechanical activation of Piezo1 drives osteoarthritis through Kdm5c‐mediated epigenetic silencing Excessive mechanical stress activates Piezo1 ion channels. The Ca^2+^ mediated by Piezo1 promote mechanotransduction of the cytoskeleton and cause the nucleus deformation as well as the downregulation of H3K4me3. Meanwhile, the histone demethylase Kdm5c removes H3K4me3 modifications on the cartilage synthesis genes *Col2a1* and *Runx3* promoters, thereby silencing their expression. Telmisartan has the potential for immediate clinical translation as a direct Kdm5c inhibitor.

This study delineates a complete signaling axis from mechanical sensing at the plasma membrane to epigenetic silencing in the nucleus, establishing the Piezo1–Kdm5c pathway as a fundamental driver of OA. We demonstrate that mechanical stress activates Piezo1, triggering calcium influx and cytoskeleton‐mediated force transmission that results in significant dilation of nuclear pores and chromatin remodeling. The histone demethylase Kdm5c erases the active histone mark H3K4me3 at promoters of key cartilage‐anabolic genes such as *Col2a1* and *Runx3*, ultimately disrupting cartilage homeostasis. The translational potential of this pathway is underscored by drug repurposing: we identify the clinical drug telmisartan as a direct Kdm5c inhibitor that effectively blocks this axis and ameliorates OA progression across cellular, murine, and human model systems (Figure 8). Our findings reposition Piezo1 from a mere mechanosensitive ion channel to a central signaling hub coordinating nuclear mechano‐responses. While previous studies have focused on Piezo1‐mediated calcium signaling and its downstream metabolic and inflammatory effects [9, 10], our work expands this view by showing that Piezo1‐transmitted forces are conveyed directly to the nucleus, with the nuclear pore complex acting as a key effector in this process. This aligns with the emerging concept of the nucleus as a dynamic mechanosensory organelle [11, 12]. We propose that Piezo1 activation may directly or indirectly alter nucleoporin conformation or interactions, thereby transforming nuclear pores from static conduits into mechanoresponsive gateways. This force‐regulated nucleocytoplasmic transport mechanism offers a new paradigm for understanding how physical signals directly impinge on the epigenome.

To further dissect the causal relationship between Piezo1‐activated Ca^2^^+^ signaling and the downstream cytoskeletal‐nuclear deformation, we performed BAPTA‐AM pretreatment prior to mechanical loading. Notably, BAPTA‐AM completely abrogated the stress‐induced F‐actin reorganization, including both the directional alignment of stress fibers along the tensile axis and the formation of the perinuclear actin cap. Concomitantly, the nuclear enlargement and increased membrane curvature were also fully reversed to static control levels. These results demonstrate that Piezo1‐mediated Ca^2^^+^ influx is an indispensable upstream signal for cytoskeletal remodeling and subsequent nuclear deformation. Mechanistically, Ca^2^^+^ may activate downstream effectors such as calpain and the RhoA/ROCK pathway, which orchestrate actin dynamics and force transmission from the plasma membrane to the nucleus [2, 11]. While we do not exclude the possibility that direct physical tension transmitted via the cytoskeleton also contributes to nuclear deformation, our BAPTA‐AM experiments unequivocally establish the essential role of Ca^2^^+^ signaling in this mechanotransduction cascade. These findings align with recent studies demonstrating that heterochromatin‐driven nuclear softening protects the genome against mechanical stress [11], and that calcium channels like TRPV2 mediate cytoskeletal‐nuclear coupling in other mechanosensitive cell types [2].

Multiple histone demethylases have been implicated in the complex epigenetic landscape of OA [20, 21, 22]. For example, LSD1 (KDM1A) contributes to OA pathogenesis via H3K9 demethylation [23, 24, 25], while KDM6B and KDM7A/B modulate cartilage homeostasis through regulation of H3K27me3 and H3K79me2, respectively [26, 27, 28, 29, 30]. Our study introduces Kdm5c into this network with a unique role: it is the only demethylase shown to be mechanically regulated by Piezo1 and to specifically target H3K4me3. As H3K4me3 is a canonical marker of active promoters, its loss directly correlates with transcriptional silencing [31]. Thus, Kdm5c‐mediated erosion of H3K4me3 provides a unified epigenetic mechanism through which mechanical stress precisely suppresses cartilage anabolic gene expression, positioning Kdm5c as a more specific therapeutic target than upstream mechano‐sensors. While nuclear pore dilation is inherently non‐selective and could theoretically facilitate the entry of various nuclear factors, we propose that Kdm5c plays a dominant role in this mechano‐epigenetic axis due to two key features: its substrate specificity for H3K4me3 and its marked upregulation at both mRNA and protein levels upon mechanical stress. Other factors that may enter the nucleus under these conditions are less likely to directly target the promoters of cartilage‐anabolic genes such as *Col2a1* and *Runx3* in a specific manner. Regarding the potential upstream regulation of Kdm5c expression, our RNA‐seq data suggest that mechanical stimulation may activate certain transcriptional regulators. It is plausible that transcription factors such as YAP/TAZ or NF‐κB are involved, as previous studies have shown that mechanosensitive channels like Piezo1 can drive unique transcriptional profiles, and that stress fiber‐generated tension can regulate YAP nuclear localization, while NF‐κB is also activated by mechanical signals in osteoarthritic chondrocytes [19, 26]. However, these possibilities remain speculative and require direct experimental validation in future studies.

Repurposing telmisartan based on this mechanism represents a pivotal translational advance. We demonstrate that telmisartan's efficacy in our models stems from direct binding and inhibition of Kdm5c, stabilizing H3K4me3 levels independently of its canonical angiotensin II receptor blockade [32, 33]. Given the protracted and high‐risk trajectory of novel OA drug development [34, 35, 36, 37], leveraging telmisartan—a drug with an established clinical safety profile—offers an expedited route to clinical validation for OA therapy. An important safety consideration regarding the translational potential of telmisartan is its well‑known antihypertensive effect when administered orally. However, in the present study, telmisartan was delivered via intra‑articular injection, a route that largely restricts the drug to the joint cavity. The systemic bioavailability of intra‐articularly administered drugs is typically below 5%, which is far lower than the concentration required to elicit systemic pharmacological effects [38]. Moreover, the dose used in our mouse model (5 mg kg^−1^, intraarticular) is substantially lower than the reported oral antihypertensive dose of telmisartan (approximately 10 mg/kg/day) [39]. Therefore, it is highly unlikely that the intra‑articular regimen employed here would produce clinically relevant changes in systemic blood pressure.

Several limitations of our study warrant further investigation. First, the precise molecular mechanism underlying force‐induced nuclear pore dilation, such as potential phosphorylation of nucleoporins by mechano‐activated kinases downstream of Piezo1, remains to be elucidated. Second, the mechanisms by which Kdm5c accesses chromatin targets following nuclear pore dilation remain unclear, its potential cooperation with specific importins merits further exploration. Third, while we focused on H3K4me3, whether the Piezo1–Kdm5c axis coordinately regulates other OA‐relevant histone marks, such as H3K27me3 [24], represents a compelling future direction.

Notwithstanding these limitations, our work offers several conceptual and practical advances. To our knowledge, this is the first study to outline a complete causal pathway from pathological mechanical sensing Piezo1 through nuclear pore remodeling to specific epigenetic erasure Kdm5c‐H3K4me3, establishing a mechano‐epigenetic cascade as a fundamental driver of OA. Our findings open new therapeutic vistas: targeting the nuclear pore–epigenetic interface may represent a novel strategy for developing disease‐modifying OA drugs, and the role of Kdm5c as a mechanoresponsive epigenetic eraser may extend beyond OA to other mechanopathologies such as cardiovascular fibrosis [17]. Finally, the success of telmisartan repositioning underscores the power of integrating computational drug screening with mechanobiology to rapidly identify clinically actionable therapies [40, 41].

## 4 Conclusions

In conclusion, we defined a coherent Piezo1–Kdm5c mechano‐epigenetic axis that physically and functionally links the extracellular mechanical environment to transcriptional regulation in the nucleus. Our work not only reveals a new OA pathogenesis pathway but also establishes a foundational framework for targeting epigenetic regulators downstream of mechanical stress, with telmisartan representing a promising and readily translatable candidate for therapeutic intervention.

## 5 Experimental Section

### 5.1 Study Design

The primary objective of this study was to elucidate the mechanism by which Piezo1‐mediated mechanotransduction affects OA pathogenesis via H3K4me3 regulation in chondrocytes. The investigation utilized articular cartilage specimens from patients with OA and varus deformity (poor lower limb force lines), a mouse model with chondrocyte‐specific conditional knockout of Piezo1 (*Col2a1^CreERT^; Piezo1^flox/flox^*), and their littermate controls (*Piezo1^flox/flox^*). Telmisartan was administered via intra‐articular knee injection in mice, with a minimum of six animals per group. The ATDC5 cells were treated with small interfering RNA (siRNA), the Piezo1 agonist Yoda1, or telmisartan. OA was surgically induced in both the conditional knockout and control mice using a standardized model of post‐traumatic OA. Phenotypic assessment of human cartilage, mouse cartilage, and chondrocytes was performed with RNA‐sequencing and CUT&Tag sequencing, histology, atomic force microscopy (AFM), and scanning electron microscopy (SEM). Articular cartilage sections were stained with safranin O for proteoglycan visualization or processed for immunohistochemistry and immunofluorescence. All experiments were replicated independently at least three times. Experimental groups were randomly assigned, and all procedures and evaluation were performed under blinded conditions. The sample size was determined by power calculations based on known analytical variability. All samples, organisms, and participants were randomly assigned to each experimental group. Using completely randomized grouping controls covariates.

### 5.2 Human Osteoarthritis Cartilage Samples

This study was approved by the Ethics Committee of Shanghai Ninth People's Hospital, Shanghai Jiao Tong University School of Medicine (No. SH9H‐2021‐T401‐2). All patients were from the Chinese Han population. The sample size was calculated to detect a mean difference of 8 in the total Osteoarthritis Research Society International (OARSI) scores, with a standard deviation of 12; a sample size of *n* = 6 was chosen based on this power calculation. All six patients provided written informed consent, and the demographic data for all patients are detailed in Table S1. Participants were diagnosed with primary symptomatic OA with varus deformity of the lower limb, based on clinical and radiographic criteria. Those who had other forms of arthritis, such as rheumatoid arthritis, or who were taking medications that could affect bone remodeling (including estrogen and bisphosphonates) were excluded. All OA cartilage specimens were divided into three groups: mild, moderate and severe OA.

### 5.3 In Vivo Experiments

Ten‐week‐old male C57BL/6 mice were purchased from Shanghai Jihui Laboratory Animal Co., Ltd. (Shanghai, China) and maintained under a 12 h light/dark cycle with free access to food and water. *Piezo1^flox/flox^* mice and *Col2a1^CreERT^* mice were obtained from the Jackson Laboratory (Bar Harbor, ME, USA; stock #029213 and #018148, respectively). To generate chondrocyte‐specific Piezo1 knockout mice, *Piezo1^flox/flox^* mice were crossed with *Col2a1^CreERT^* mice. At 8 weeks of age, the resulting *Col2a1^CreERT^*; *Piezo1^flox/flox^* mice received intraperitoneal injections of tamoxifen (TAM, 100 mg kg^−1^ body weight; Sigma–Aldrich) for 5 consecutive days to induce Cre‐mediated recombination. Control mice (*Piezo1^flox/flox^*) were treated with the same dose of TAM on an identical schedule. All mice were housed in specific pathogen‐free conditions at the Experimental Animal Center of Shanghai Ninth People's Hospital, Shanghai Jiao Tong University School of Medicine. OA was induced by destabilization of the medial meniscus (DMM) surgery according to established protocols. For intra‐articular injections, an insulin syringe was used. The needle was inserted through the gap between the medial edge of the patella and the tibial plateau, and the drug or solvent was injected slowly. The drug concentration was standardized to 5 mg kg^−1^ body weight, with injections administered twice weekly for 4 consecutive weeks. An injection was considered successful if no fluid reflux was observed. All animal experiments were approved by the Institutional Animal Care and Use Committee of Shanghai Ninth People's Hospital (No. SH9H‐2023‐A3‐1) and performed in accordance with institutional guidelines.

### 5.4 Cell Culture and Tensile Strain

The ATDC5 cells (Fuheng Center Cell Bank, China) were cultured in DMEM (Sigma, USA) supplemented with 10% fetal bovine serum (FBS; Sigma, USA), 1% L‐glutamine (Sigma, USA), and 1% penicillin‐streptomycin (Gibco, USA). Cells were maintained in a humidified incubator at 37°C with 5% CO_2_. For mechanical stimulation experiments, cells were seeded onto flexible‐bottom culture plates (Flexcell International, USA) and allowed to adhere until reaching approximately 90% confluence. Cyclic tensile strain was then applied using a computer‐controlled bioreactor system, with parameters set at 15% elongation and 0.5 Hz sinusoidal waveform for a duration of 12 h. All experimental procedures were performed under sterile conditions at 37°C with 5% CO_2_.

### 5.5 Atomic Force Microscope

The dissected femoral samples were mounted onto a circular specimen disk for atomic force microscope (AFM). Nanoindentation testing was performed on the articular cartilage surface of the medial condyle using a silicon nitride cantilever with a nominal spring constant of approximately 0.6 N m^−1^. For each medial condyle, three distinct indentation sites were selected, and six repeated nanoindentation measurements were performed at each site. The selection of indentation sites was guided by identifying relatively flat regions on the cartilage surface using the integrated optical microscope of the AFM system. All indentations were conducted with an approach velocity of 1.98 µm s^−1^ and a maximum indentation force of approximately 30 nN. The loading segment of the force–displacement curves was analyzed using the Hertzian contact model.

### 5.6 Scanning Electron Microscope

Samples were fixed with 4% paraformaldehyde (PFA) for 24 h and then rinsed with PBS. Following rinsing, the samples were dehydrated through a graded ethanol series. After dehydration, the samples were freeze‐dried, mounted on specimen stubs with conductive adhesive, and examined for ultrastructural morphology using a field‐emission scanning electron microscope (SEM) (SU‐8010, Hitachi, Japan). Energy‐dispersive X‐ray spectroscopy (EDS) was subsequently conducted with the integrated system (X‐MaxN; Oxford Instruments, UK) on the same field of view to quantify the relative abundance and spatial distribution of the principal elements within the sample.

### 5.7 Transmission Electron Microscope

The ultrastructure of the tibial plateau cartilage tissue and ATDC5 cells was observed by transmission electron microscope (TEM). Adherent cell pellets obtained by centrifugation and cartilage explants (approximately 1 mm^3^) were fixed in glutaraldehyde and then dehydrated through a graded ethanol series. After dehydration, the samples were infiltrated with resin overnight and embedded. Following resin polymerization, ultrathin sections were prepared, stained with uranyl acetate and lead citrate, and imaged using a Talos L120C TEM (Thermo Fisher Scientific).

### 5.8 BAPTA‐AM Treatment

ATDC5 cells were incubated with 10 µM BAPTA‐AM (Selleck) in serum‐free medium for 30 min at 37°C prior to mechanical loading. BAPTA‐AM was prepared in DMSO (final DMSO concentration ≤0.1%). Control cells received DMSO vehicle only. The BAPTA‐AM‐containing medium was maintained throughout the cyclic tensile strain application.

### 5.9 Fluo‐4 AM Imaging

ATDC5 cells were seeded at a density of 5 × 10^4^ cells per well in confocal culture dishes and cultured in DMEM supplemented with 10% FBS. Prior to experimentation, cells were maintained in serum‐free medium for 12 h to ensure synchronization. For calcium imaging, cells were loaded with Fluo‐4 AM (Thermo Fisher Scientific, USA) by incubation at 37°C under 5% CO_2_ for 45 min in the dark to allow for complete probe internalization. Subsequently, cells were washed with PBS to remove extracellular free probes. The culture dish was placed on the stage of a laser scanning confocal microscope (LSM 880; Zeiss, Germany), and cells in different fields of view were scanned to record the changes in intracellular calcium ion fluorescence intensity.

### 5.10 SiRNA Transfection

ATDC5 cells were seeded in 6‐well plates at a density of 2 × 10^5^ cells well^−1^ and cultured in DMEM supplemented with 10% FBS for 48 h to reach approximately 70% confluence. For siRNA transfection, cells were transfected with 50 nM of either negative control siRNA (si‐NC) or target‐specific siRNAs (si‐Piezo1 and si‐Kdm5c) using Lipofectamine 3000 (Thermo Fisher Scientific, Waltham, MA, USA) according to the manufacturer's instructions. Transfection efficiency was verified by real‐time quantitative polymerase chain reaction (q‐PCR) and western blot experiments. The sequences of PCR primers and siRNA are respectively listed in Tables S2 and S3.

### 5.11 Alcian Blue Staining

After removing the culture medium, ATDC5 cells were gently washed with PBS to remove residual serum components. Cells were then fixed with 4% PFA at room temperature for 10 min. For alcian blue staining, cells were incubated with Alcian blue solution for 30 min at room temperature. Unbound dye was removed by thorough rinsing with PBS until the washing solution became clear. Images were captured using an inverted microscope.

### 5.12 Molecular Docking

The Kdm5c crystal structure (PDB: 4BCU) was optimized using Schrödinger's Protein Preparation Wizard. Hydrogen atoms were added, bond orders were assigned, and disulfide bonds were corrected. Protonation states were generated at pH 7.0 using PROPKA, followed by restrained energy minimization with the OPLS4 forcefield (heavy atom RMSD convergence: 0.3 Å). The drug‐repurposing compound library (L9200, Sigma) was prepared using LigPrep (Schrödinger 2021‐3). Compounds were desalted, ionized using Epik (pH 7.0 ± 2.0), and stereoisomers/tautomers were retained. Conformational sampling generated ≤32 poses per ligand. Initial standard precision (SP) docking with flexible ligands using default settings retained the top 20% of poses. This was followed by refined extra precision (XP) docking with post‐docking minimization. Compounds with XP GlideScore < −8 kcal mol^−1^ (569 initial hits) were prioritized. Further filtering retained molecules with ligand efficiency between −0.6 and −0.3 kcal mol^−1^ (271 final candidates). Protein‐ligand interaction fingerprints (PLIF), structural diversity, and binding mode analyses were performed.

### 5.13 CCK‐8 Assay

ATDC5 cells were seeded in 96‐well plates at a density of 5 × 10^3^ cells per well in 100 µL of DMEM supplemented with 10% FBS. The plates were incubated at 37°C under 5% CO_2_ for 24 h. Experimental groups were treated with complete medium containing varying drug concentrations, while the control group received an equivalent volume of drug‐free complete medium. After treatment, 10 µL of CCK‐8 reagent was added to each well, and the plates were incubated at 37°C for 1 h in the dark. The optical density (OD) at 450 nm was measured using a microplate reader.

### 5.14 Culture of Human Cartilage Explants

Human cartilage tissue samples were obtained from patients undergoing knee joint replacement surgery. Cylindrical cartilage explants were prepared using a corneal trephine to ensure standardized dimensions and minimal mechanical damage. All samples were immediately immersed in DMEM supplemented with 10% FBS and maintained at 37°C under 5% CO_2_. The culture medium was replaced every 48 h. After the culture period, explants were fixed in 4% PFA at 4°C and decalcified in 10% EDTA. Tissues were then dehydrated through a graded ethanol series, cleared in xylene, and embedded in paraffin. Serial coronal sections were cut using a rotary microtome and mounted on adhesive slides.

### 5.15 Real‐Time Quantitative PCR

Total RNA was isolated from samples using TRIzol reagent (Invitrogen, USA). RNA concentration and purity were determined using a Nanodrop spectrophotometer (Thermo Fisher Scientific, USA). cDNA was synthesized from total RNA using a reverse transcription kit (TaKaRa, Japan) under the following conditions: 37°C for 15 min, followed by 85°C for 5 s. qPCR was performed using the SYBR Premix kit (TaKaRa, Japan) on a Roche LightCycler 480 system. The reaction program consisted of 40 cycles of 95°C for 5 s and 60°C for 30 s. All primer sequences are detailed in the Supplementary Tables and all primers were synthesized by Sangon Biotech (Shanghai, China).

### 5.16 Western Blotting

Samples were collected and lysed on ice for 30 min using RIPA buffer containing protease and phosphatase inhibitors. After centrifugation at 12 000 ×g at 4°C for 15 min, the supernatant was collected, mixed with loading buffer, and boiled for 10 min. Proteins were separated by electrophoresis on precast gels at 80 V until entering the separating gel, then at 120 V until the dye front reached the bottom of the gel. Proteins were transferred to PVDF membranes (Millipore, USA). Membranes were blocked with 5% non‐fat milk in tris‐buffered saline with 0.1% Tween‐20 (TBST) and incubated with diluted primary antibody at 4°C overnight. After washing with TBST, membranes were incubated with an HRP‐conjugated secondary antibody for 1 h, washed again, and visualized using an ECL chemiluminescence kit. The grayscale values of target bands were normalized to an internal reference protein and analyzed using ImageJ software.

### 5.17 Immunofluorescence

Cells were seeded onto confocal culture dishes and treated as required. After treatments, the culture medium was aspirated and cells were washed gently with PBS. Cells were fixed with 4% PFA permeabilized with 0.5% Triton X‐100 for 10 min, and blocked with 5% bovine serum albumin (BSA) for 30 min. Cells were incubated with diluted specific primary antibody at 4°C overnight, followed by incubation with fluorophore‐conjugated secondary antibodies for 1 h in the dark. Nuclei were counterstained with DAPI. Imaging was performed using a laser‐scanning confocal microscope.

### 5.18 Nuclear Morphology Measurement

Nuclear size was quantified from confocal images of Lamin A/C‐stained nuclear membranes. For each chondrocyte, the nuclear membrane signal was optimized by noise reduction and contrast enhancement. Threshold segmentation and morphological operations were applied, and then “Analyze Particles” function was used to extract continuous contours, excluding fragments smaller than 5 µm^2^. The projected area and equivalent diameter were recorded. Nuclear curvature analysis assessed local nuclear membrane deformation. A spline ROI was manually drawn along the nuclear membrane, and eccentricity was calculated through elliptic fitting (values closer to 1 indicate a flatter shape). Local curvature was calculated at 1 µm intervals using the Kappa Curve plugin, and the average curvature was exported. Differences between groups were tested by ANOVA.

### 5.19 Analysis of Nuclear Pore

The size quantification of the nuclear pore complex (NPC) was based on clear transmission electron microscopy images of nuclear membrane cross‐sections. First, the nuclear membrane contour was identified using threshold segmentation in ImageJ. Then, a spline ROI was manually drawn along the nuclear membrane, and the grayscale curve of the vertical section was extracted. The diameter of the nuclear pore was defined by the trough width between adjacent peaks. For elliptical deformation caused by oblique sectioning, the length of the short axis was measured as the true aperture after correction by the elliptical fitting tool. Excluding pores with a cross‐section angle greater than 30°, the final output was the average diameter.

### 5.20 Analysis of Chromatin

Chromatin ratio analysis focuses on the nuclear region. First, using the ImageJ software, all TEM images are preprocessed, with the contrast adjusted uniformly and converted into an 8‐bit grayscale image. Subsequently, the boundaries of each cell nucleus are manually delineated to determine the region of interest for analysis. Based on the typical bimodal distribution characteristics of the gray histogram, we adopt a multi‐level threshold segmentation method to classify pixels according to the difference in chromatin electron density: the area with the highest electron density and the darkest staining (with a gray value ≤ 50) was defined as heterochromatin, the area with medium electron density and relatively lighter staining (with a gray value between 51 and 120) was defined as euchromatin, and the extremely bright area with a gray value > 120 (usually corresponding to nuclear pores, nucleoli or intranuclear spaces) was excluded from the chromatin analysis. After threshold setting, the pixel areas of euchromatin and heterochromatin are calculated separately. The proportion of euchromatin is calculated using the formula “(euchromatin area / total chromatin area) × 100%”.

### 5.21 Immunohistochemistry

Tissue samples were fixed with 4% PFA, decalcified with EDTA decalcifying solution, dehydrated through graded ethanol series, cleared with xylene, and embedded in paraffin before sectioning. Sections were incubated with H_2_O_2_ at room temperature, subjected to antigen retrieval with citrate buffer, blocked with 5% BSA, and then incubated with primary antibodies at 4°C overnight. HRP‐labeled secondary antibody was added and incubated at room temperature for 30 min. Stained sections were visualized using a slide scanner.

### 5.22 RNA Sequencing

Chondrocytes were isolated from the knee articular cartilage of *Piezo1^flox/flox^*; *Col2a1^CreERT^* mice and *Piezo1^flox/flox^* mice. Isolated chondrocytes were divided into four experimental groups: Control–Static, Control–Stress, Conditional Knockout–Static, and Conditional Knockout–Stress. Total RNA was extracted using TRIzol reagent (Thermo Fisher Scientific Inc., USA). Sequencing library preparation and subsequent high‐throughput RNA sequencing were performed by OeBiotech Corporation (Shanghai, China).

### 5.23 CUT&Tag

CUT&Tag was performed using the CUT&Tag Assay Kit (53160, Active Motif, China) according to the manufacturer's instructions. Library preparation and sequencing were performed by Shanghai Oebiotech Co., Ltd. Briefly, 2 × 10^5^ cells per sample were collected and bound to magnetic beads, incubated for 3 h with H3K4me3 primary antibody (Active Motif), and further incubated with secondary antibody for 1 h. After three washes, cells were incubated for 1 h with pA‐Tn5. Samples were resuspended in tagmentation buffer for 1 h. DNA was then extracted, purified, amplified by PCR, and processed into sequencing libraries.

### 5.24 ChIP‐PCR

ChIP assays were performed using the ChIP‐IT Express Kit (Active Motif) according to the manufacturer's protocol. Briefly, ATDC5 cells (approximately 2 × 10^6^ per sample) were cross‐linked with 1% formaldehyde for 10 min at room temperature, followed by quenching with 0.125 M glycine for 5 min. Cells were washed twice with ice‐cold PBS, scraped, and collected by centrifugation. Cell pellets were lysed and nuclei were isolated using the provided lysis buffers. Chromatin was sheared by sonication to an average fragment size of 200–600 bp. After centrifugation, the sheared chromatin supernatant was diluted with ChIP dilution buffer. An aliquot (10 µl) was saved as “input” for each sample. For immunoprecipitation, 2 µg of anti‐H3K4me3 antibody or normal mouse IgG was added to each chromatin sample and incubated overnight at 4°C with rotation. Protein G magnetic beads (provided in the kit) were added and incubated for 3 h at 4°C. Beads were washed sequentially with low‐salt, high‐salt, and LiCl wash buffers, followed by two washes with TE buffer. Immune complexes were eluted with elution buffer, and cross‐linking was reversed by adding 5 M NaCl and proteinase K, followed by incubation at 65°C for 4 h. DNA was purified using the provided spin columns and eluted in 50 µl of elution buffer. Quantitative PCR was performed using SYBR Green Master Mix (TaKaRa, Japan) on a Roche LightCycler 480 system, with primers specific for the promoter regions of Col2a1 and Runx3 (sequences listed in Table S4). Enrichment was calculated as percentage of input relative to a standard curve. All experiments were performed with three independent biological replicates.

### 5.25 Statistical Analysis

All data were derived from at least three independent biological replicates or repeated measurements, with specific sample sizes (*n* = 3 or 6 as indicated in each figure legend) used for each analysis. No data transformation or normalization was applied. Outliers were assessed by the Grubbs' test (α = 0.05) and excluded if identified; no extreme outliers were detected in the final datasets. All data are presented as mean ± standard deviation (SD). Normality and homogeneity of variances were assessed using the Shapiro‐Wilk test and Brown‐Forsythe test, respectively. For comparisons between two groups, a two‐tailed unpaired Student's *t*‐test was used for independent samples, and a two‐tailed paired Student's t‐test was used for paired samples. For multiple comparisons, one‐way analysis of variance (ANOVA) followed by Tukey's post hoc test was applied when data satisfied both normality and equal‑variance assumptions; otherwise, the Kruskal‑Wallis test followed by Dunn's post hoc test was used. For two‑factor experimental designs, two‑way ANOVA followed by Tukey's post hoc test was performed. Correlation analyses were performed using Pearson's correlation coefficient for normally distributed data or Spearman's rank correlation for non‑normally distributed data. A significance level (α) of 0.05 was set for all statistical tests, and a *p* value less than 0.05 was considered statistically significant. All statistical analyses and graphical representations were carried out using GraphPad Prism 9.0 (GraphPad Software, San Diego, CA, USA).

## Author Contributions

**T.K**. and **X.L**.: conceptualization, investigation, formal analysis, and writing – original draft. **Y.W**. and **L.S**.: investigation and formal analysis. **J.W**. and **H.W**.: investigation and formal analysis. **J.C**.: methodology and formal analysis. **Y.Y**., **K.Y**., **L.W**. and **L.C**.: investigation. **H.L**.: methodology and supervision. **M.Y**. and **Z.Y**.: conceptualization, supervision, funding acquisition, and writing – review and editing.

## Ethics Statement

This study was approved by the Ethics Committee of Shanghai Ninth People's Hospital, Shanghai Jiao Tong University School of Medicine (No. SH9H‐2021‐T401‐2). All animal experiments were approved by the Institutional Animal Care and Use Committee of Shanghai Ninth People's Hospital (No. SH9H‐2023‐A3‐1) and performed in accordance with institutional guidelines.

## Conflicts of Interest

The authors declare no conflicts of interest.

## Supporting information




**Supporting File**: advs76089‐sup‐0001‐SuppMat.docx.

## Data Availability

The data that support the findings of this study are available from the corresponding author upon reasonable request.
