# Supplementary material for: Mechanical Activation of Piezo1 Drives Osteoarthritis Through Kdm5c‐Mediated Epigenetic Silencing
Source: Adv Sci (Weinh). 2026 Jun 12:e76089. Online ahead of print. doi: 10.1002/advs.76089 (PMC13336863; doi:10.1002/advs.76089)
Supplement: Supplementary file 1 — Supporting file: advs76089‐sup‐0001‐SuppMat.docx [file ADVS-9999-e76089-s001.docx]

**Supplementary Figures**


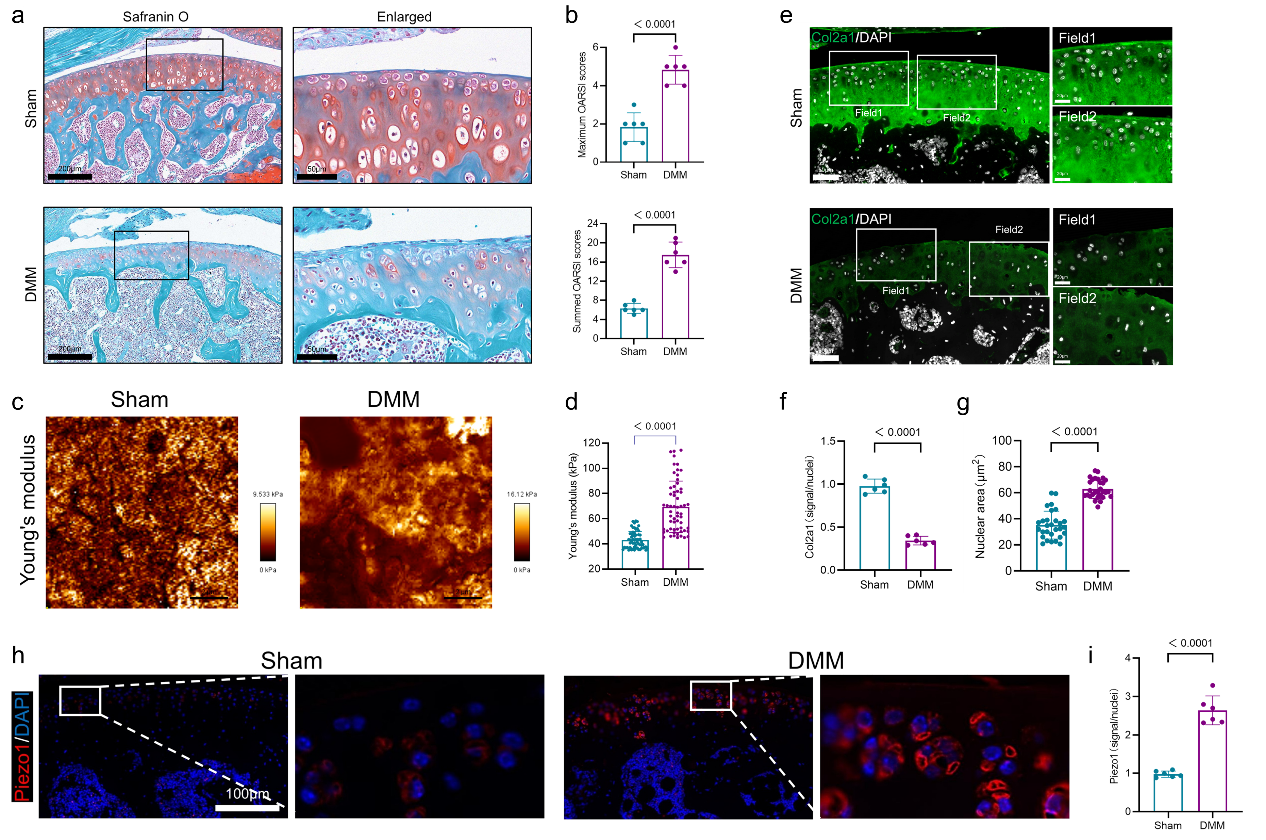


**Supplementary Figure 1** Mechanical response and nuclear deformation of chondrocytes in DMM mice cartilage a) Representative Safranin O staining of cartilage in Sham and DMM mice. Scale bars: 100 and 50 μm. b) Maximum OARSI scores and summed OARSI scores of Sham and DMM mice (n = 6). c) Mechanical scanning heatmaps of the surface from OA cartilage samples from the unstressed side and the stressed side. Scale bars: 2 μm. d) Young's modulus of cartilage in Sham and DMM mice (n=60 samples from six mice). e) Immunofluorescence of Col2a1 and DAPI of Sham and DMM mice. Scale bars: 50 and 20 μm. f) Quantification of Col2a1 fluorescence intensity of Sham and DMM mice (n = 6). g) Chondrocyte nuclear area of OA cartilage samples from Sham and DMM mice (n = 30 samples from six Sham or DMM mice). h) Immunofluorescence of Piezo1 and DAPI of Sham and DMM mice. Scale bars: 100 μm. i) Relative fluorescence intensity of Piezo1 of Sham and DMM mice (n = 6). The p value is indicated on the statistical graph. Values are means ± SDs. Comparison between two groups was performed by a two-tailed Student’s t-test.


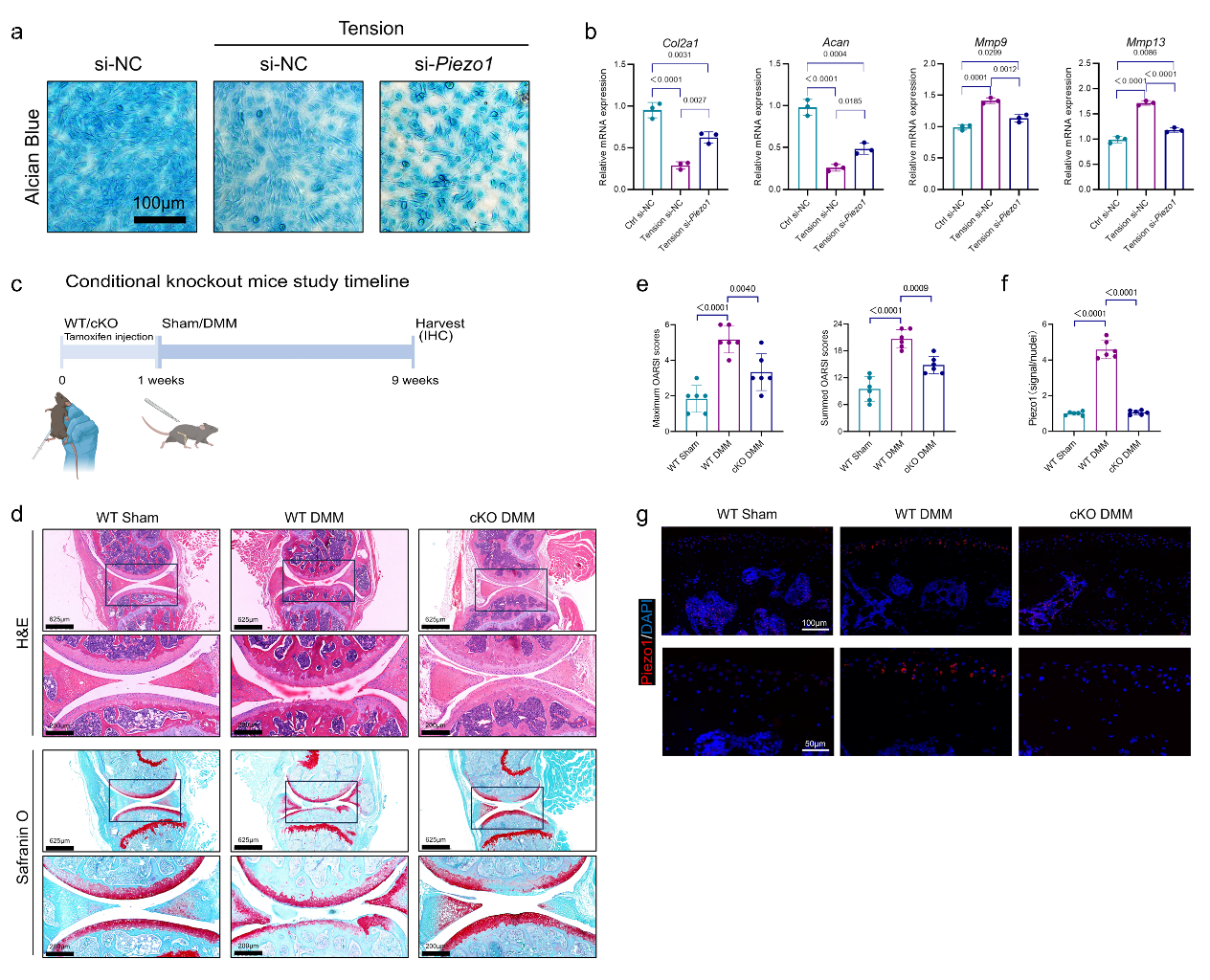


**Supplementary Figure 2** Genetic deletion of piezo1 attenuates osteoarthritis phenotypes a) Alcian blue staining of ATDC5 cells of si-NC Ctrl, si-NC Tension and si-*Piezo1* Tension groups. Scale bars: 100 μm. b) mRNA expression levels of *Col2a1*, *Acan*, *Mmp9* and *Mmp13* of si-NC Ctrl, si-NC Tension and si-*Piezo1* Tension groups (n=3). c) Representative H&E and Safranin O staining of WT Sham, WT DMM and cKO DMM groups. Scale bars: 625 and 200 μm. d) Maximum OARSI scores and summed OARSI scores of WT Sham, WT DMM and cKO DMM groups (n = 6). e) Quantification of Piezo1 immunofluorescence intensity of WT Sham, WT DMM and cKO DMM groups (n = 6). f) Relative fluorescence intensity of Piezo1 of WT Sham, WT DMM and cKO DMM groups (n = 6). g) Immunofluorescence of Piezo1 and DAPI of WT Sham, WT DMM and cKO DMM groups. Scale bars: 100 and 50 μm. The p value is indicated on the statistical graph. Values are means ± SDs. Multiple comparison was performed by one-way one-way ANOVA with Tukey’s post-hoc analysis.


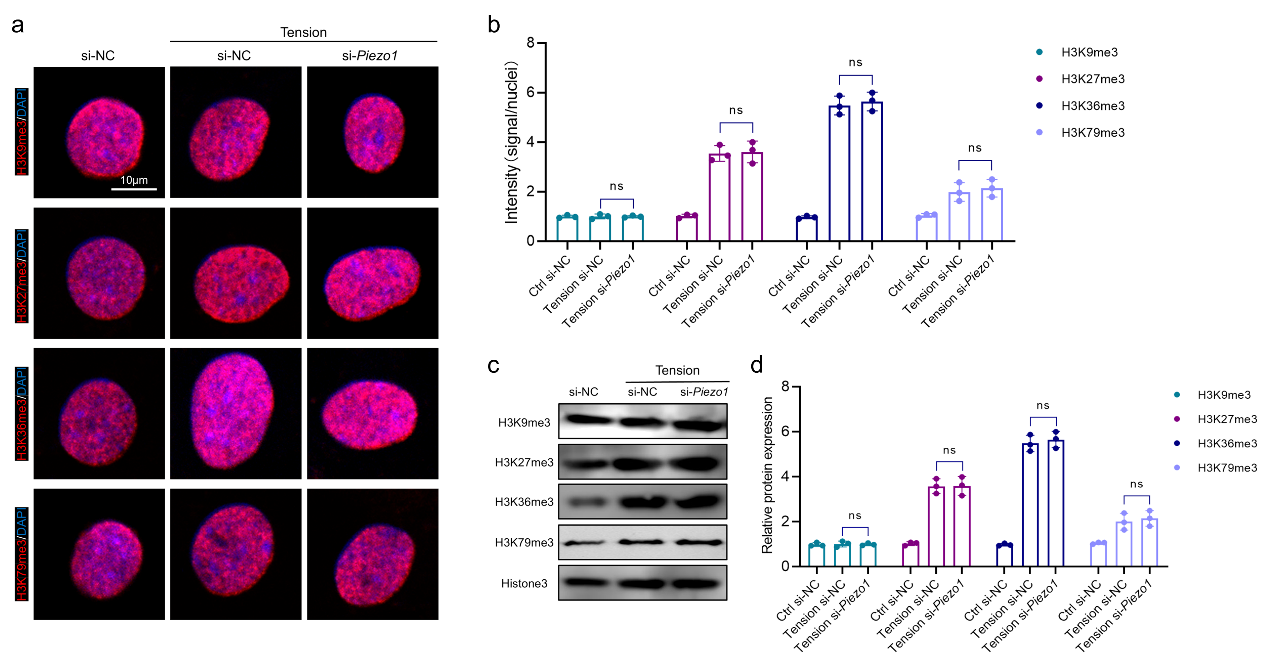


**Supplementary Figure 3** Piezo1-mediated mechanical stress on histone methylation in ATDC5 cells a) Immunofluorescence of H3K9me3, H3K27me3, H3K36me3, H3K79me3 and DAPI of si-NC Ctrl, si-NC Tension and si-*Piezo1* Tension groups. Scale bars: 10 μm. b) Quantification of fluorescence intensity of H3K9me3, H3K27me3, H3K36me3 and H3K79me3 of si-NC Ctrl, si-NC Tension and si-*Piezo1* Tension groups (n = 3). c) The protein expression levels of H3K9me3, H3K27me3, H3K36me3 and H3K79me3 of si-NC Ctrl, si-NC Tension and si-*Piezo1* Tension groups. d) Relative protein expression of H3K9me3, H3K27me3, H3K36me3 and H3K79me3 of si-NC Ctrl, si-NC Tension and si-*Piezo1* Tension groups (n = 3). The p value is indicated on the statistical graph. Values are means ± SDs. Multiple comparison was performed by one-way one-way ANOVA with Tukey’s post-hoc analysis.


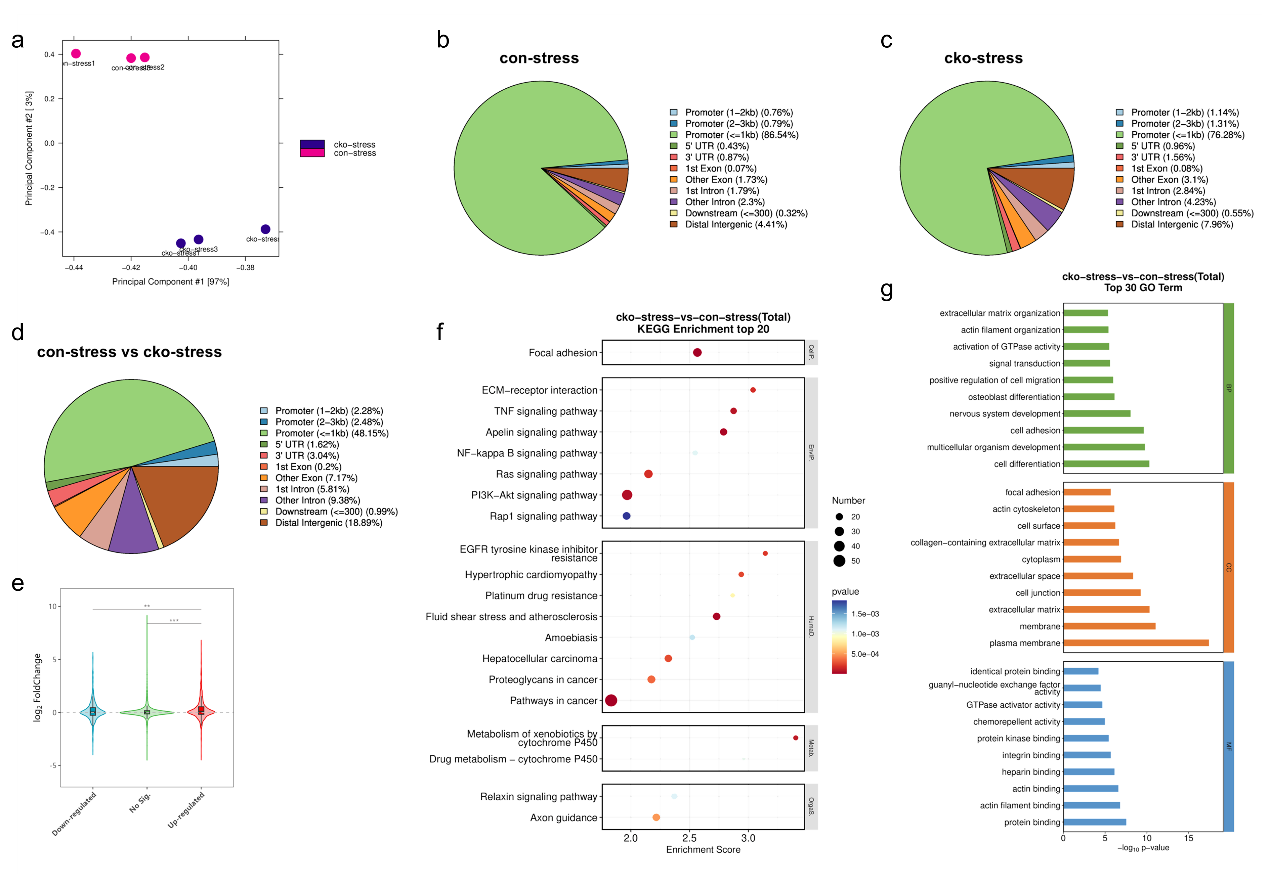


**Supplementary Figure 4** Genome-Wide Profiling of H3K4me3 by CUT&Tag in Piezo1-mediated mechanical responses of chondrocytes a) Principal component analysis (PCA) of the con-stress and cko-stress groups. b) Genome-wide distribution of H3K4me3 on the genome in con-stress group. c) Genome-wide distribution of H3K4me3 on the genome in cko-stress group. d) Genome-wide distribution of H3K4me3 on the genome after the intersection of con-stress and cko-stress groups. e) Distribution of H3K4me3 on the genome of differential genes between con-stress and cko-stress groups. f) KEGG pathway enrichment analysis of the differentially expressed genes between con-stress and cko-stress groups. g) GO term enrichment analysis of the differentially expressed genes between con-stress and cko-stress groups.


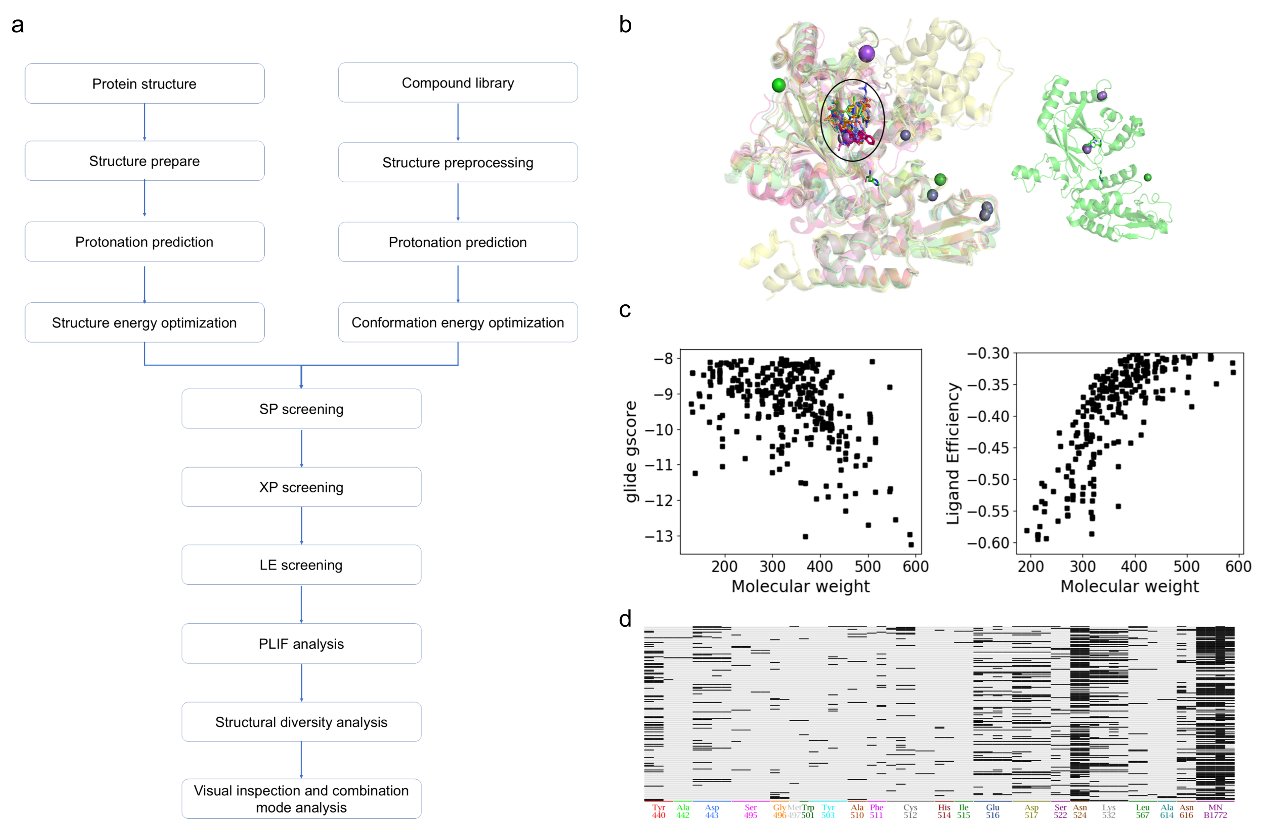


**Supplementary Figure 5** Molecular docking and drug screening for Kdm5c a) Schematic of the molecular docking-based screening workflow for Kdm5c inhibitors. b) Molecular docking score in the molecular weight of the screening result. c) Ligand efficiency in the molecular weight of the screening results. d) Molecular interaction fingerprint analysis


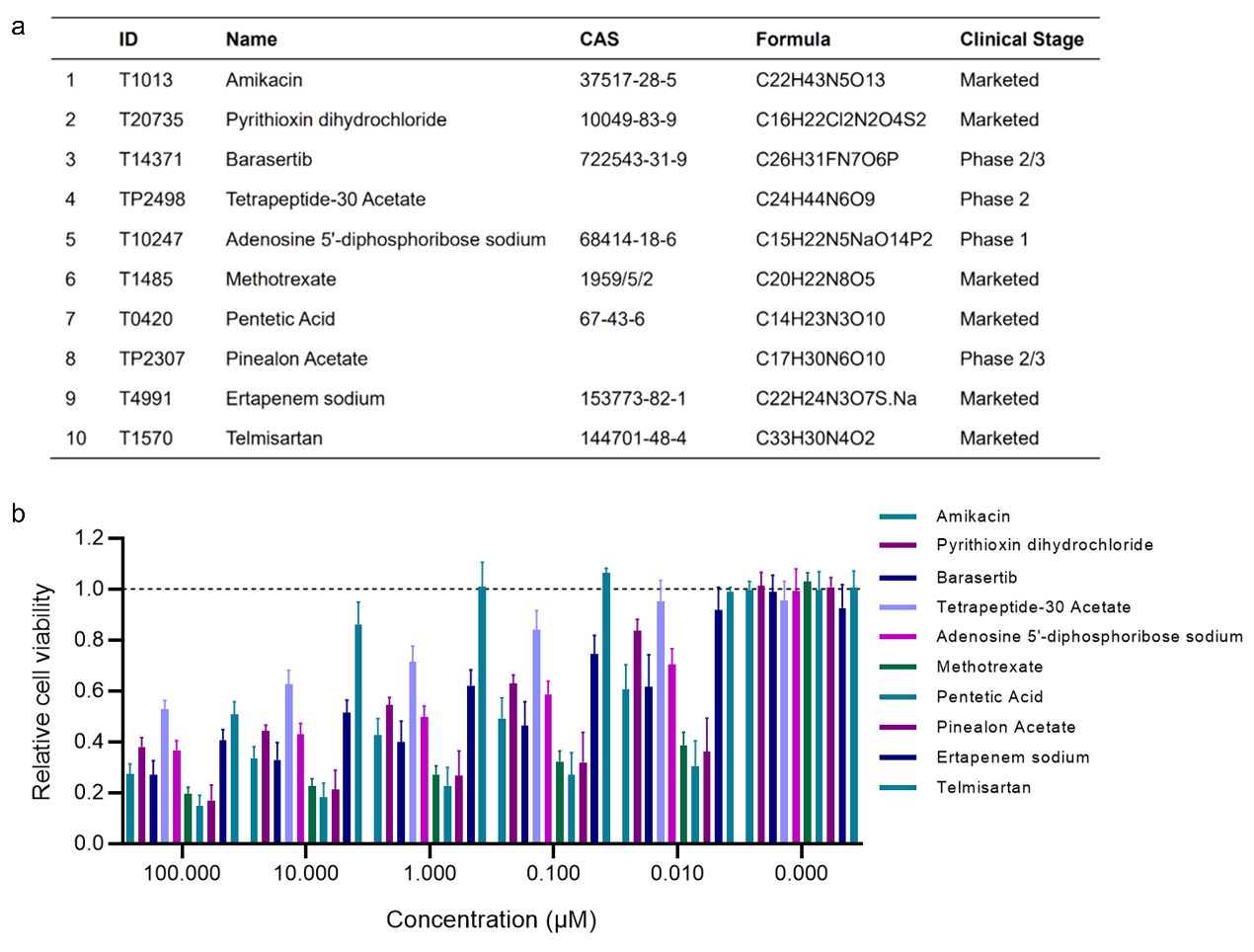


**Supplementary Figure 6** The top 10 candidate drug screening a) The top 10 candidate compounds ranked by docking score. b) The toxicity of the top 10 candidate drugs to ATDC5 cells (n=3).


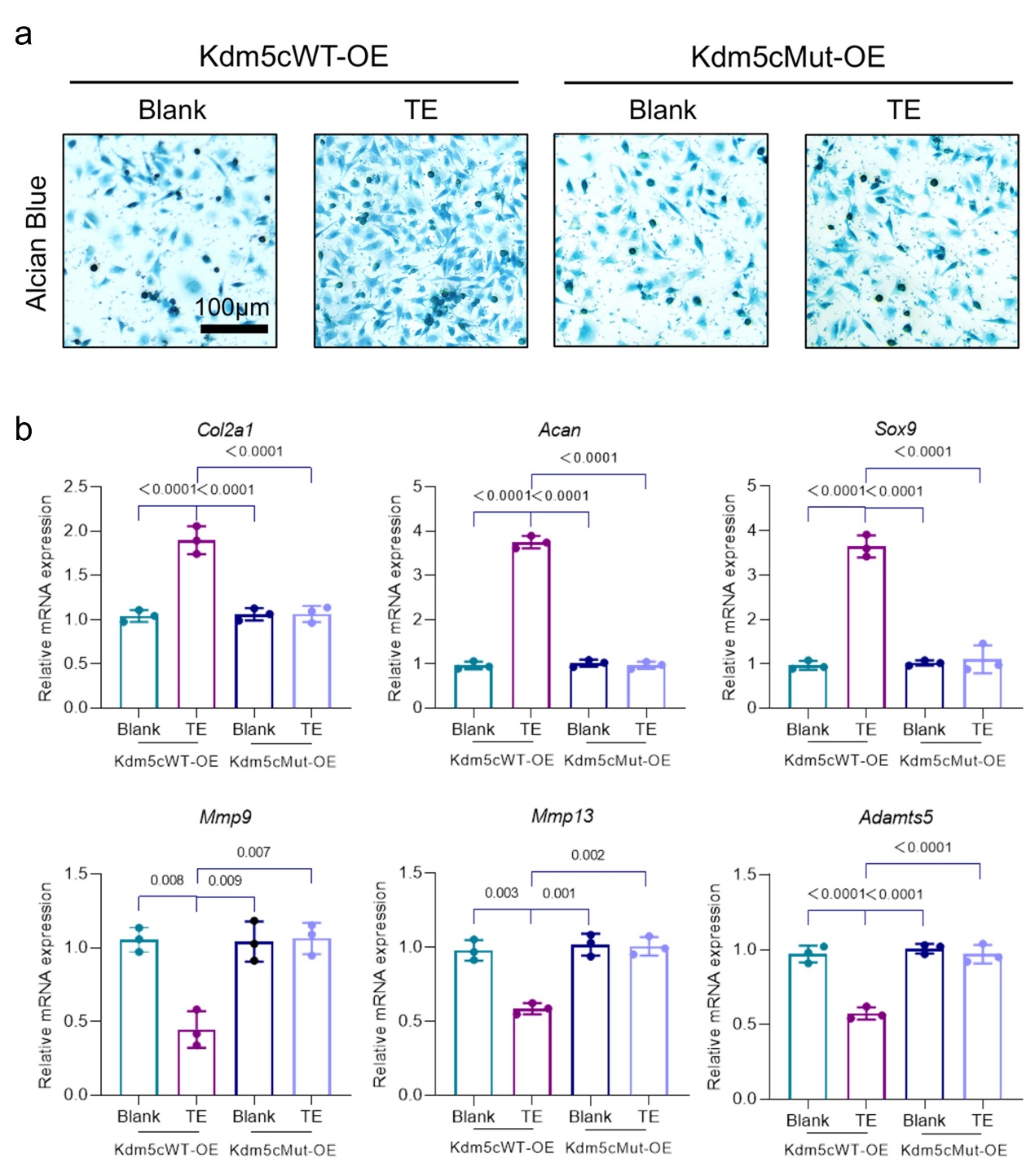


**Supplementary Figure 7** The therapeutic effect of telmisartan on ATDC5 cells of overexpressing mutant Kdm5c a) Alcian blue staining of glycosaminoglycans in mechanically loaded ATDC5 cells of Kdm5cWT-OE and mutant Kdm5cMut-OE. **b)** The mRNA expression levels of *Col2a1*, *Acan*, *Sox9*, *Mmp9*, *Mmp13* and *Adamts5* in mechanically loaded ATDC5 cells overexpressing of Kdm5cWT-OE and mutant Kdm5cMut-OE (n=3). The p value is indicated on the statistical graph. Values are means ± SDs. Multiple comparison was performed by one-way one-way ANOVA with Tukey’s post-hoc analysis.

**Supplementary Tables**

**Table 1. The demographic data**

| **ID** | **Age** | **Sex** | **Knee deformity** | **Kellgren-Lawrence (KL) grade** |
| --- | --- | --- | --- | --- |
| 1 | 76 | F | varum | 3 |
| 2 | 79 | M | varum | 4 |
| 3 | 68 | M | valgus | 4 |
| 4 | 72 | F | varum | 3 |
| 5 | 77 | F | varum | 4 |
| 6 | 82 | F | varum | 4 |

**Table 2. Real-time PCR primers**

| **Target gene** | **Forward 5’-3’** | **Reverse 5’-3’** |
| --- | --- | --- |
| *Col2a1* | GCTACACTCAAGTCACTGAACAACCA | TCAATCCAGTAGTCTCCGCTCTTCC |
| *Acan* | TCGAGGACAGCGAGGCC | TCGAGGGTGTAGCGTGTAGAGA |
| *Sox9* | AGGAAGCTGGCAGACCAGTA | GGTGGTCCTTCTTGTGCTGC |
| *Mmp9* | CTGGACAGCCAGACACTAAAG | CTCGCGGCAAGTCTTCAGAG |
| *Mmp13* | TGGAGTAATCGCATTGTGAGAGTC | CCAGCCACGCATAGTCATATAGATAC |
| *Adamts5* | TGGGACAGTGTGGCTGTGTA | ACACGGTTGTCATCGTCCTC |
| *Gapdh* | ATGGTGAAGGTCGGTGTGAA | TGAGTGGAGTCATACTGGAACA |
| *COL2* | GGCAATAGCAGGTTCACGTACA | CGATAACAGTCTTGCCCCACTT |
| *ACAN* | TCGAGGACAGCGAGGCC | TCGAGGGTGTAGCGTGTAGAGA |
| *MMP13* | ACTGAGAGGCTCCGAGAAATG | GAACCCCGCATCTTGGCTT |
| *ADAMTS5* | TGCGGCAAACAGATCCAGA | CCACAGTCTTGGCACTCCAT |
| *GAPDH* | GGAGCGAGATCCCTCCAAAAT | GGCTGTTGTCATACTTCTCATGG |

**Table 3. siRNA sequence**

| **siRNA** | **Forward 5’-3’** | **Reverse 5’-3’** |
| --- | --- | --- |
| si-NC | UUCUCCGAACGUGUCACGUTT | ACGUGACACGUUCGGAGAATT |
| si-*Piezo1* 1 | GCUAUCAGACACCAUUUAUTT | AUAAAUGGUGUCUGAUAGCTT |
| si-*Piezo1* 2 | CCAGGCUAUUUGGUCUCAATT | UUGAGACCAAAUAGCCUGGTT |
| si-*Piezo1* 3 | GCUCACAUUCUGGCUCCUUTT | AAGGAGCCAGAAUGUGAGCTT |
| si-*Kdm5c* 1 | CGCAUUGUUUAUCCCUAUGAAdTdT | UUCAUAGGGAUAAACAAUGCGdTdT |
| si-*Kdm5c* 2 | AGCAAGCUACCCGGGAAUAUAdTdT | UAUAUUCCCGGGUAGCUUGCUdTdT |
| si-*Kdm5c* 3 | GCCCAAUAUCCAGUCUCUCAAdTdT | UUGAGAGACUGGAUAUUGGGCdTdT |

**Table 4. ChIP-PCR primers**

| **Target gene** | **Forward 5’-3’** | **Reverse 5’-3’** |
| --- | --- | --- |
| ChIP-*Col2a1* | ACTGAGCTCGAAACGTCCTG | CTTTCTCAGGTCCCTGCGTT |
| ChIP-*Runx3* | TGGCCGCTACAACTGAAGAG | AGCGGGGGACATCTCTTAGT |

**Table 5. Antibodies**

| **Antibodies** | **Source** | **Identifier** |
| --- | --- | --- |
| Collagen Type II Polyclonal antibody | Proteintech | Cat No. 28459-1-AP |
| SOX9 Polyclonal antibody | Proteintech | Cat No. 55152-1-AP |
| Anti-RUNX3 antibody | Abcam | ab135248 |
| GAPDH Polyclonal antibody | Proteintech | Cat No. 10494-1-AP |
| Anti-FAM38A/PIEZO1 | Abcam | ab128245 & ab324311 |
| Anti-Lamin A + Lamin C | Abcam | ab133256 |
| Anti-F-actin antibody | Abcam | ab205 |
| KDM5C Polyclonal antibody | Proteintech | Cat No. 14426-1-AP |
| Histone H3K4me3 antibody | Proteintech | Cat No : 91263 |
| Histone H3K9me3 antibody | Proteintech | Cat No : 39285 |
| Histone H3K27me2me3 antibody | Proteintech | Cat No : 39536 |
| Histone H3K36me3 antibody | Proteintech | Cat No : 61021 |
| Anti-Histone H3 (tri methyl K79) antibody | Abcam | ab251493 |
| Histone H3 Polyclonal antibody | Proteintech | Cat No. 17168-1-AP |
| Mouse IgG antibody | Proteintech | Cat No. B900620 |
| AF488-labeled Goat Anti-Rabbit IgG (H+L) | Beyotime | A0423 |
| AF488-labeled Goat Anti-Mouse IgG (H+L) | Beyotime | A0428 |
| AF555-labeled Donkey Anti-Rabbit IgG (H+L) | Beyotime | A0453 |
| AF555-labeled Donkey Anti-Mouse IgG (H+L) | Beyotime | A0460 |
